# Heterogeneity and transmission of food safety-related enterotoxigenic *Staphylococcus aureus* in pig abattoirs in Hubei, China

**DOI:** 10.1128/spectrum.01913-23

**Published:** 2023-09-29

**Authors:** Zhihao Zhu, Simin Wu, Xingyu Chen, Wei Tan, Geng Zou, Qi Huang, Xianrong Meng, Dong-Liang Hu, Shaowen Li

**Affiliations:** 1 Key Laboratory of Preventive Veterinary Medicine in Hubei Province, College of Veterinary Medicine, Huazhong Agricultural University, Wuhan, China; 2 Department of Zoonoses, Kitasato University School of Veterinary Medicine, Towada, Japan; Agriculture and Agri-Food Canada, Lacombe, Canada

**Keywords:** *Staphylococcus aureus*, food safety, heterogeneity, transmission, genomic epidemiology

## Abstract

The dissemination of *Staphylococcus aureus* in the pork production chain is a major food safety concern. Abattoirs can serve both as disruptor and transmitter for *S. aureus*. In this study, we conducted a systematic genomic epidemiology research on the prevalence, heterogeneity, and transmission of *S. aureus* in 3,638 samples collected from four pig abattoirs in Hubei province, China. Our findings revealed substantial heterogeneity between *S. aureus* recovered from samples collected at upstream (from stunning step to head-removal step) and downstream (from splitting step to chilling step) locations within the slaughter process. Overall, 966 (26.6%) samples were positive for *S. aureus*, with significantly higher overall prevalence for upstream samples (29.0%, 488/1,681) compared to downstream samples (24.4%, 478/1,957). Antimicrobial susceptibility testing demonstrated that the isolates from the upstream exhibited significantly higher resistance proportions to different antimicrobials than those from the downstream. Whole-genome sequencing of 126 isolates revealed that ST398 (32.9%, 23/70) and ST9 (22.9%, 16/70) were more common among upstream isolates, while ST7 (35.7%, 20/56) and ST97 (28.6%, 16/56) were most frequently observed among downstream isolates. Additionally, molecular characterization analysis demonstrated that upstream isolates possessed significantly higher enterotoxigenic potential, more antimicrobial resistance genes, and *S. aureus* pathogenicity islands than downstream isolates. Notably, we discovered that enterotoxigenic *S. aureus* could be transmitted across different slaughter stages, with knives, water, and air serving as vectors. Although slaughtering processes had a substantial effect on reducing the food safety risk posed by enterotoxigenic *S. aureus*, the possibility of its widespread transmission should not be disregarded.

**IMPORTANCE:**

*Staphylococcus aureus* (*S. aureus*) is one of the most important foodborne pathogens, and can cause foodborne poisoning by producing enterotoxins. Pork is a preferable reservoir and its contamination often occurs during the slaughter process. Our findings revealed significant differences in the prevalence, antimicrobial resistance, and enterotoxigenic potential between the upstream and downstream isolates within the slaughter process. Also, it is imperative not to overlook enterotoxigenic *S. aureus* transmitted across all stages of the slaughter process, with notable vectors being knives, water, and air. These findings hold significant implications for policy-makers to reassess their surveillance projects, and underscore the importance of implementing effective control measures to minimize the risk of *S. aureus* contamination in pork production. Moreover, we provide a more compelling method of characterizing pathogen transmission based on core-SNPs of bacterial genomes.

## INTRODUCTION

Foodborne illnesses pose a constant threat to public health and socioeconomic development worldwide. *Staphylococcus aureus* (*S. aureus*), a major foodborne pathogen, produces multiple virulence factors and enterotoxins that cause human infectious diseases and food poisoning (1). As of now, a total of 29 types of staphylococcal enterotoxins (SEs) or staphylococcal enterotoxin-like (SEl) toxins have been identified (2). The overuse of antimicrobials for the prevention of animal diseases and growth promotion has resulted in a high prevalence of multidrug resistance (MDR), as reported in animal *S. aureus* isolates (3). In particular, the transmission of livestock-associated methicillin-resistant *S. aureus* (LA-MRSA) to humans via meat and meat products constitutes a serious food safety burden (4). The dominant lineage of LA-MRSA in pigs varies by geographic location, with ST398 in Europe (5) and ST9 in most Asian countries (6), and zoonotic transmission of LA-MRSA has been reported for some lineages, particularly ST9, ST398, and ST88 (7 – 9).

China is the largest pork producer and consumer worldwide. Pork can serve as a primary reservoir for *S. aureus*, and slaughter practices are essential to interrupt the transmission of foodborne pathogens to humans (10). Direct or indirect contact with animals is the main predisposing factor for human colonization by LA-MRSA strains (11, 12). The contamination of pork and carcasses by LA-MRSA has been widely reported (13, 14), and the transmission of MRSA during the pork production chain has been well documented (15, 16), but the characterization of transmission of *S. aureus* still lacks clarity.

Over the last decade, whole-genome sequencing (WGS) has revolutionized molecular epidemiology (17). It is a high throughput method to provide in-depth genomic information on pathogens, and bioinformatic analysis on genomes from WGS has been included in epidemiological research to characterize pathogen transmission (18, 19). Importantly, a growing array of bioinformatics tools is being developed, which will soon make WGS more readily applicable to microbial epidemiology (20).

In this study, to investigate the changes in the prevalence of enterotoxigenic *S. aureus* and identif the transmission pathways of *S. aureus* within abattoirs, we assessed the prevalence of *S. aureus* in four pig abattoirs located in Hubei province, China, during 2019. A total of 543 isolates were subjected to antimicrobial susceptibility testing, and 126 isolates underwent WGS. Then, we analyzed the differences between isolates from upstream and downstream of slaughter process, characterized the heterogeneity of antimicrobial resistance genes (ARGs), virulence factors (VFs), and mobile genetic elements (MGEs), and demonstrated the transmission of enterotoxigenic *S. aureus* isolates within abattoirs via core-SNPs (single nucleotide polymorphisms). Our study highlighted the significant changes in the prevalence of enterotoxigenic *S. aureus* and identified the transmission pathways of *S. aureus* within abattoirs. Our findings will offer valuable insights for policy-makers in the development of risk monitoring program and management strategy for *S. aureus*.

## RESULTS

### Occurrence of *S. aureus* within abattoirs

*S. aureus* was found in 966 (26.6%, 95% CI: 25.1–28.0) of the total 3,638 samples, collected at the four abattoirs. Table 1; Table S1 indicate that the overall prevalence of *S. aureus* in upstream of slaughter practice (488/1,681, 29.0%) was significantly higher than that in downstream (478/1957, 24.4%) (OR: 1.266; *P* = 0.002). Across different abattoirs, the overall prevalence of *S. aureus* was significantly higher in abattoir D (97/266, 36.5%) compared to abattoirs A (OR: 0.697; *P* = 0.013), B (OR: 0.590; *P* = 0.000), and C (OR: 0.515; *P* = 0.000). Furthermore, the overall prevalence of *S. aureus* was significantly higher in abattoir A compared to abattoir C (OR: 1.355; *P* = 0.006). Among the sample collections, the prevalence of nasal swabs after head removal (NSAHR) (238/599, 39.7%) was the most frequent (*P* < 0.005), followed by pork after chilling (PAC) (75/221, 33.9%) and nasal swabs after stunning (NSAS) (181/542, 33.4%), while carcass swabs after dehairing (CSAD) (69/540, 12.8%) was the least frequent (*P* < 0.05). Among environmental samples, the prevalence of *S. aureus* varied from 9.1% (WS, 1/11) to 67.9% (AD, 19/28).

**TABLE 1:** Overview of *S. aureus* prevalence among abattoirs^*a*^

	A		B		C		D		Total	
	Prevalence (%)	95% CI	Prevalence (%)	95% CI	Prevalence (%)	95% CI	Prevalence (%)	95% CI	Prevalence (%)	95% CI
Upstream										
NSAS	27.3, 42/154	20.4–35.0	28.8, 69/240	23.1–34.9	52.5, 63/120	43.2–61.7	25.0, 7/28	10.7–44.9	33.4, 181/542	29.4–37.5
CSAD	20.6, 37/180	14.9–27.2	2.5, 6/240	0.9–5.4	21.6, 26/120	14.7–30.1	ns	-	12.8, 69/540	10.1–15.9
NSAHR	22.9, 43/188	17.1–29.5	57.1, 137/240	50.6–63.4	25.0, 30/120	17.5–33.7	54.9, 28/51	40.3–68.9	39.7, 238/599	35.8–43.8
Total	23.37, 122/522	19.8–27.2	29.4, 212/720	26.1–32.9	33.1, 119/360	28.2–38.2	44.3, 35/79	33.1–55.9	29.0, 488/1,681	26.9–31.3
Downstream										
CSAS	34.6, 65/188	27.8–41.8	22.1, 53/240	17.0–27.9	13.3, 16/120	7.8–20.7	10.0, 6/60	3.8–20.5	23.0, 140/608	19.7–26.6
PAS	35.2, 32/91	25.4–45.9	14.2, 17/120	8.5–21.7	17.5, 21/120	11.2–25.5	6.7, 2/30	0.8–22.1	19.9, 72/361	15.9–24.4
CSAC	26.9, 50/186	20.7–33.9	19.1, 45/236	14.3–24.7	5.0, 6/120	1.9–10.6	63.2, 36/57	49.3–75.6	22.9, 137/599	19.6–26.4
PAC	49.2, 30/61	36.1–62.3	22.5, 27/120	15.4–31.0	ns	-	45.0, 18/40	29.3–61.5	33.9, 75/221	27.7–40.6
Total	33.7, 177/526	29.6–37.9	19.8, 142/716	17.0–22.9	11.9, 43/360	8.8–15.8	33.2, 62/187	26.5–40.4	24.4, 478/1,957	22.5–26.4
Environment										
AD	37.5, 3/8	-	80.0, 8/10	-	80.0, 8/10	-	ns	-	67.9, 19/28	-
WS	25.0, 1/4	-	0.0, 0/5	-	0.0, 0/2	-	ns	-	9.1, 1/11	-
GS	ns	-	33.3, 9/27	-	8.3, 1/12	-	ns	-	25.6, 10/39	-
KS	ns	-	28.6, 24/84	-	0.0, 0/6	-	ns	-	26.7, 24/90	-
Overall	28.6, 303/1,060	25.9–31.4	25.3, 395/1,562	23.1–27.5	22.8, 171/750	19.8–26.0	36.5, 97/266	30.7–42.6	26.6, 966/3,638	25.1–28.0

^*a*^ ns: not sampled. “-”: not statistically significant. CI: Confidence Intervals. A, B, C, and D: abattoirs in this study. NSAS, nasal swabs after stunning; CSAD, carcass swabs after dehairing; NSAHR, nasal swabs after head-removal; CSAS, carcass swabs after splitting; PAS, pork after splitting; CSAC, carcass swabs after chilling; PAC, pork after chilling; AD, air deposition samples; WS, water samples; GS, ground swabs; KS, knives swabs.

### Antimicrobial resistance profiles

**Fig 1:**
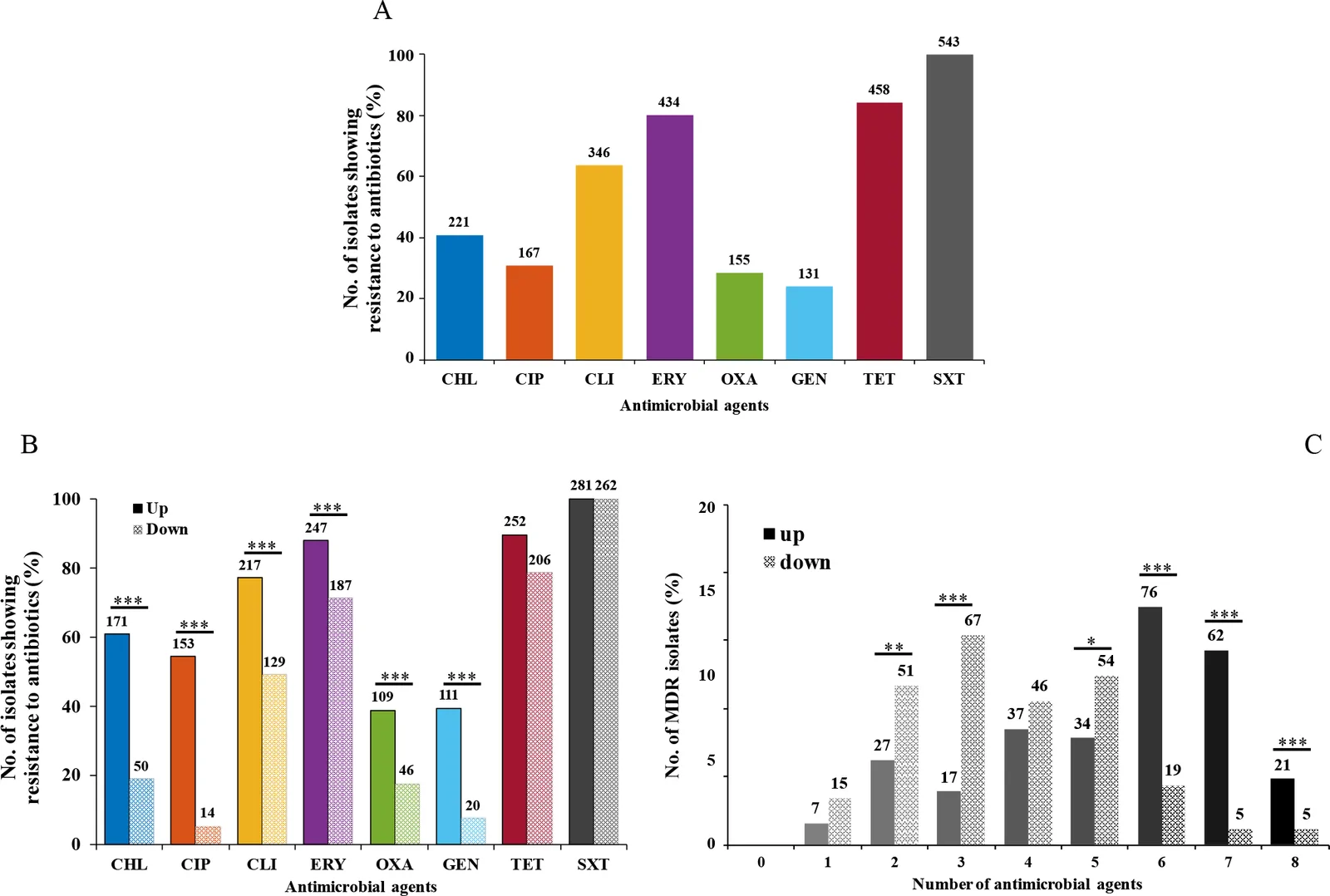
(**A**) The prevalence of 543 isolates for each antimicrobial agent. Antimicrobial agents are color-coded. The digits indicate the number of resistance isolates. (**B**) Difference of antimicrobial resistance proportion between upstream and downstream in abattoir based on 543 isolates. *P < 0.05; **P < 0.01; ***P < 0.001. (**C**) Distribution of multidrug-resistant (MDR) isolates based on 543 isolates.

A total of 543 *S*. *aureus* isolates from various sample sources were chosen for antibiotic susceptibility testing, with 3–5 isolates selected from each source. The results revealed that all isolates showed resistance to sulfamethoxazole-trimethoprim (SXT), and the resistance proportion to tetracycline (TET) and erythromycin (ERY) was 84.4% (458/543) and 79.9% (434/543), respectively, while only 24.1% (131/543) of the isolates exhibited resistance to gentamicin (GEN) (Fig. 1A). Additionally, upstream isolates showed significantly higher resistance proportions in chloramphenicol (CHL), ciprofloxacin (CIP), clindamycin (CLI), erythromycin (ERY), oxacillin (OXA), and GEN than downstream isolates (Fig. 1B). Among all 543 isolates, 81.6% (443/543) exhibited MDR to three or more classes of antimicrobial agents, with upstream isolates demonstrated a higher proportion of MDR than their downstream counterparts (Fig. 1C). The 543 isolates exhibited 44 distinct antimicrobial resistance (AMR) patterns, with CHL-CIP-CLI-ERY-GEN-SUL-TET (57/281, 20.3%) being the most frequent among upstream isolates, while ERY-SUL-TET (51/262, 19.5%) was predominant among downstream isolates (Table S2).

### Heterogeneity of sequence types and enterotoxigenic potential between the isolates of upstream and downstream

**Fig 2:**
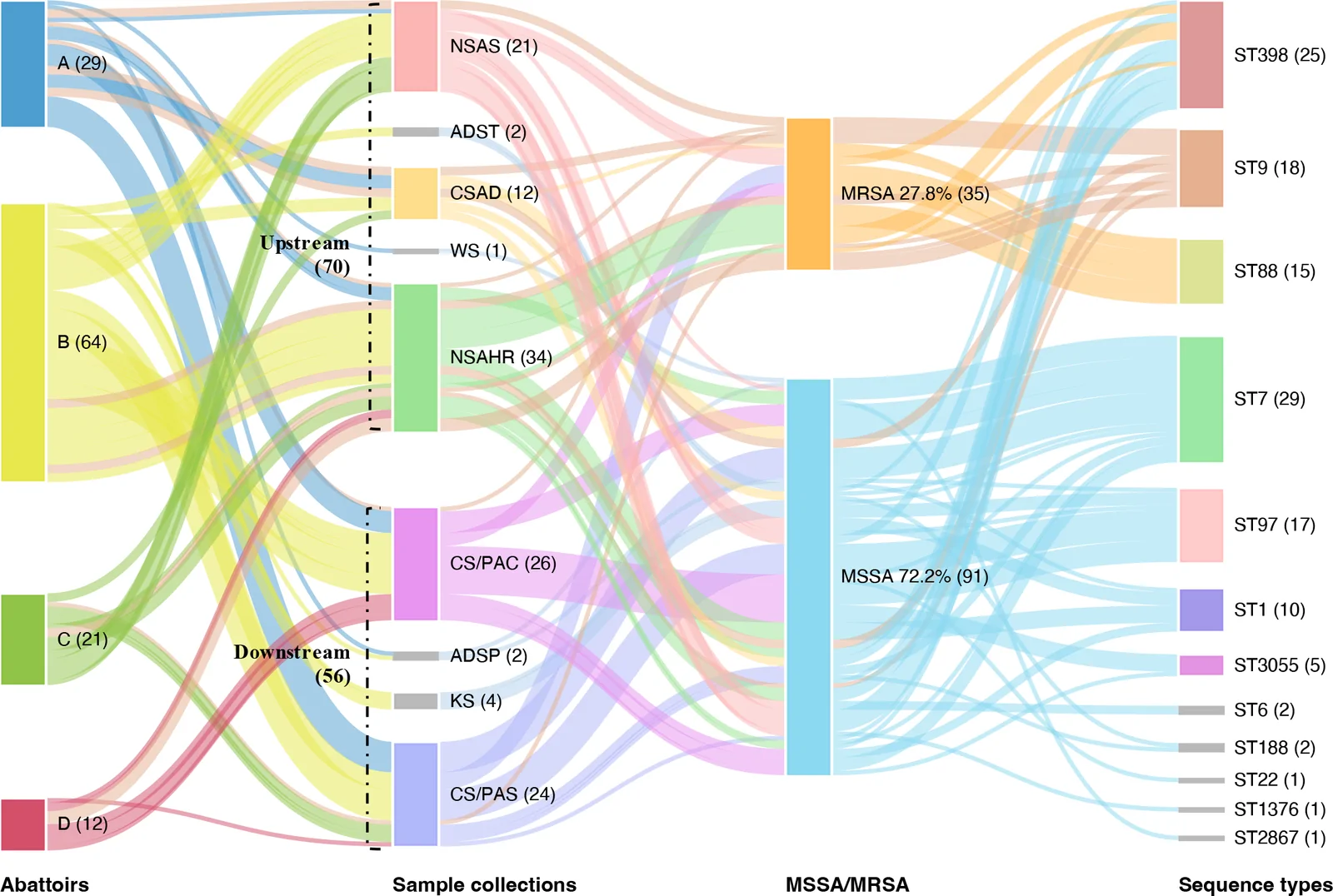
Sankey diagram combining the abattoirs, sample collections, methicillin-resistant *S. aureus* (MRSA)/methicillin-susceptible *S. aureus* (MSSA), and MLSTs (multi-locus sequence types ) of 126 *S*. *aureus*. A, B, C and D: abattoirs in this study. NSAS, nasal swabs after stunning; ADST, air deposition samples of stunning; CSAD, carcass swabs after dehairing; WS, water samples; NSAHR, nasal swabs after head-removal; CS/PAS, carcass swabs and pork after splitting; ADSP, air deposition samples of splitting; LSTKS, knives swabs; CS/PAC, carcass swabs and pork after chilling.

A total of 126 *S*. *aureus* from different sample collections were sequenced for subsequent analysis, with AMR patterns used as a reference. Figure 2; Table S3 showed that all 126 isolates (29, 64, 21, and 12 for abattoirs A, B, C, and D, respectively) displayed 12 distinct sequence types (STs). ST398 (23/70, 32.9%) and ST9 (16/70, 22.9%) were more prevalent among upstream isolates, while ST7 (20/56, 35.7%) and ST97 (16/56, 28.6%) were more frequent in downstream. MRSA (35/126, 27.8%) was only present in ST9, ST88, and ST398. Furthermore, ST398-t1451 (11/70, 15.7%) and ST88-t3622-IVc(2B) (7/70, 10.0%) were common genotypes in upstream, whereas ST7-t091 (16/56, 28.6%) and ST97-t267 (14/56, 25.0%) were more frequent in downstream.

**Fig 3:**
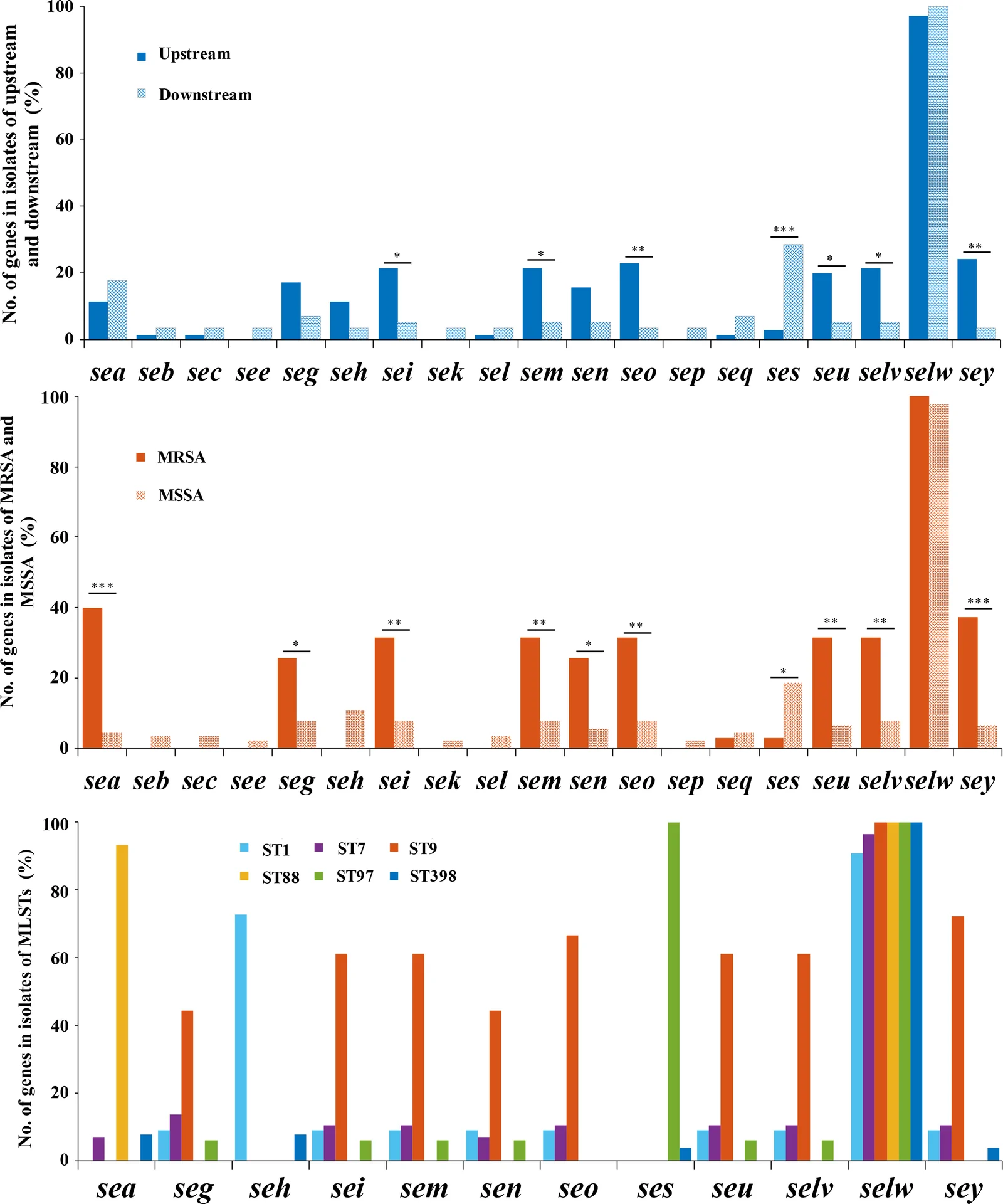
Difference of enterotoxin genes harbored among upstream and downstream (**A**) methicillin-resistant *S. aureus* (MRSA) and methicillin-susceptible *S. aureus* (MSSA) (**B**) and MLSTs (**C**) based on 126 isolates. *P < 0.05; **P < 0.01; ***P < 0.001.

A total of 19 SE/SEl genes were screened to further analyze. Upstream isolates showed significantly higher rates of carrying *sei*, *sem*, *seo*, *seu*, *selv, and sey* compared to downstream isolates (*P* < 0.05) (Fig. 3A). MRSA showed a significantly higher carrying rate of *sea*, *seg*, *sei*, *sem*, *sen*, *seo*, *seu*, *selv*, and *sey* than methicillin-susceptible *S. aureus* (MSSA) (*P* < 0.05) (Fig. 3B). Among MLSTs, ST9-*sei-seg-sem-sen-seo-seu-selv-selw-sey*, ST88*-sea-selw*, ST97*-ses-selw*, and ST1*-seh-selw* were the dominant types of SE/SEl among all isolates (Fig. 3C).

### Heterogeneity of genomic features between the isolates of upstream and downstream

**Fig 4:**
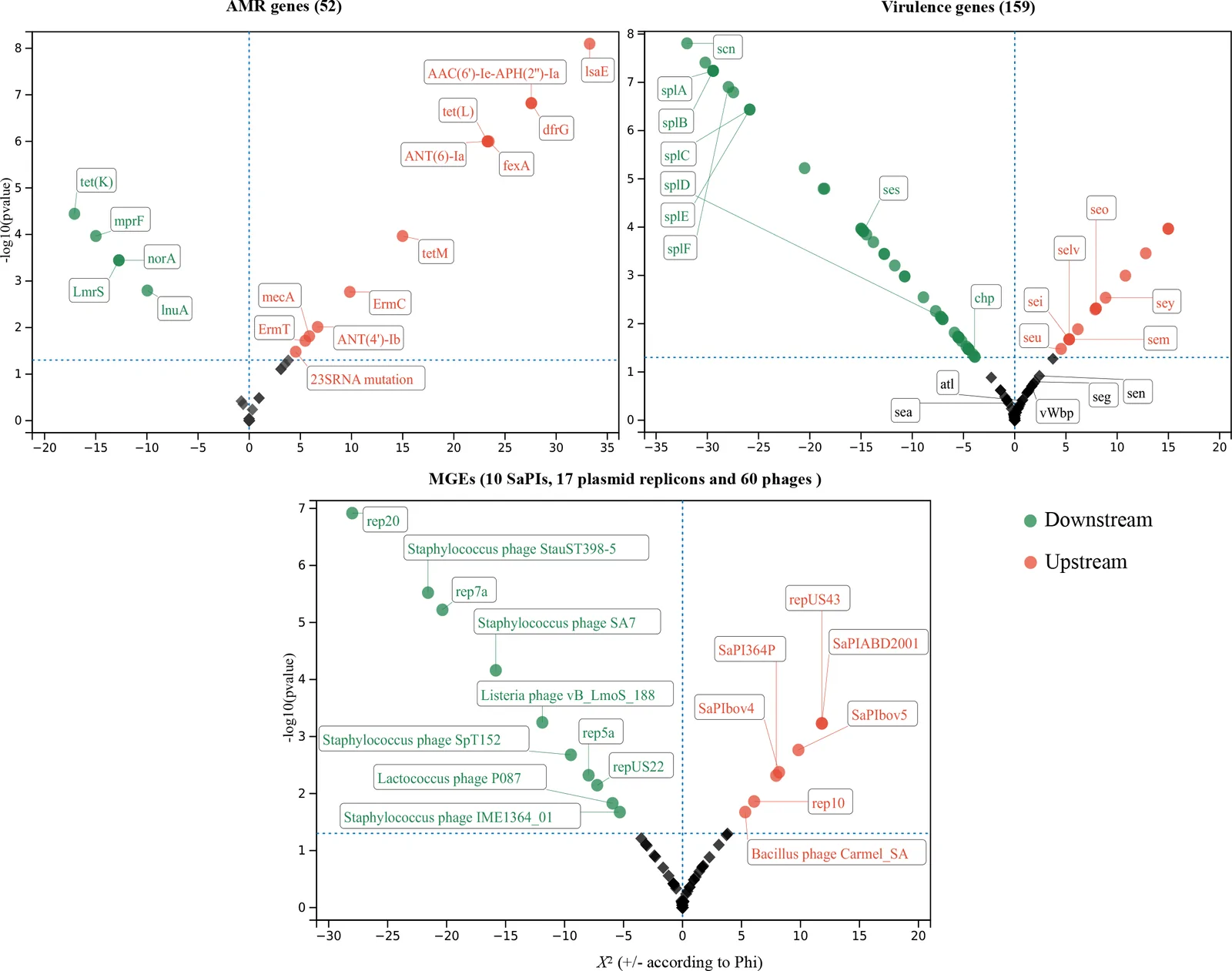
Heterogeneity of *S. aureus* in upstream and downstream for antimicrobial resistance (AMR) genes, virulence genes, and mobile genetic elements (MGEs). Statistical significance was evaluated by Chi-squared test with phi and Cramer’s V tests, the positive or negative of *Χ* ^2^ was determined by phi test.

We examined the heterogeneity of enterotoxigenic *S. aureus* in terms of ARGs (52), VFs (159), and MGEs (10 SaPIs, 17 plasmid replicons, and 60 phages) (Fig. S1 to S3) between isolates recovered from upstream and downstream. As illustrated in Fig. 4, the upstream isolates harbored more ARGs, SE/SEl genes, and SaPIs. Among the ARGs, *fexA*, *lsaE*, *ermC*, *mecA*, *ant (4')-Ib*, *aac (6')-Ie-APH (2'')-Ia*, *ant (6)-Ia*, *tetL*, and *tetM* were associated with higher resistance to CHL, CLI, ERY, OXA, GEN, and TET in the upstream isolates. Among VFs, upstream isolates showed a greater potential for enterotoxigenic due to the high carrying rate of *sei, sem, seo, seu, selv,* and *sey*. Among MGEs (Fig. S4), plasmids harbored most mobile ARGs, including tetracycline resistance gene *tetK* and *tetL*, macrolides resistance gene *ermC* and *msrA*, β-Lactam resistance gene *mecA* and *blaZ*, quinolones resistance gene *ileS* and lincomycin resistance gene *lunA*. SaPIs363P encoding *seg, sei, sem, sen, seo*, and *spl-*family genes had the highest prevalence (18.3%, 23/126), but there was no significant difference between upstream and downstream isolates (*P* > 0.05). SaPIABD2001 (22/126, 17.5%) encoded *tetM*, SaPIbov5 (20/126, 15.9%) encoded *vWbp* and *scn*, and SaPI364P (18/126, 14.3%) encoded *seg, sei, sem, sen, seo,* and *selv*. Phages may mediate the transmission of a small number of virulence genes, such as *chp, scn, atl, sea, luKF-PV*, and *luKS-PV*.

### Transmission of enterotoxigenic *S. aureus* among different slaughtering stages in abattoirs

**Fig 5:**
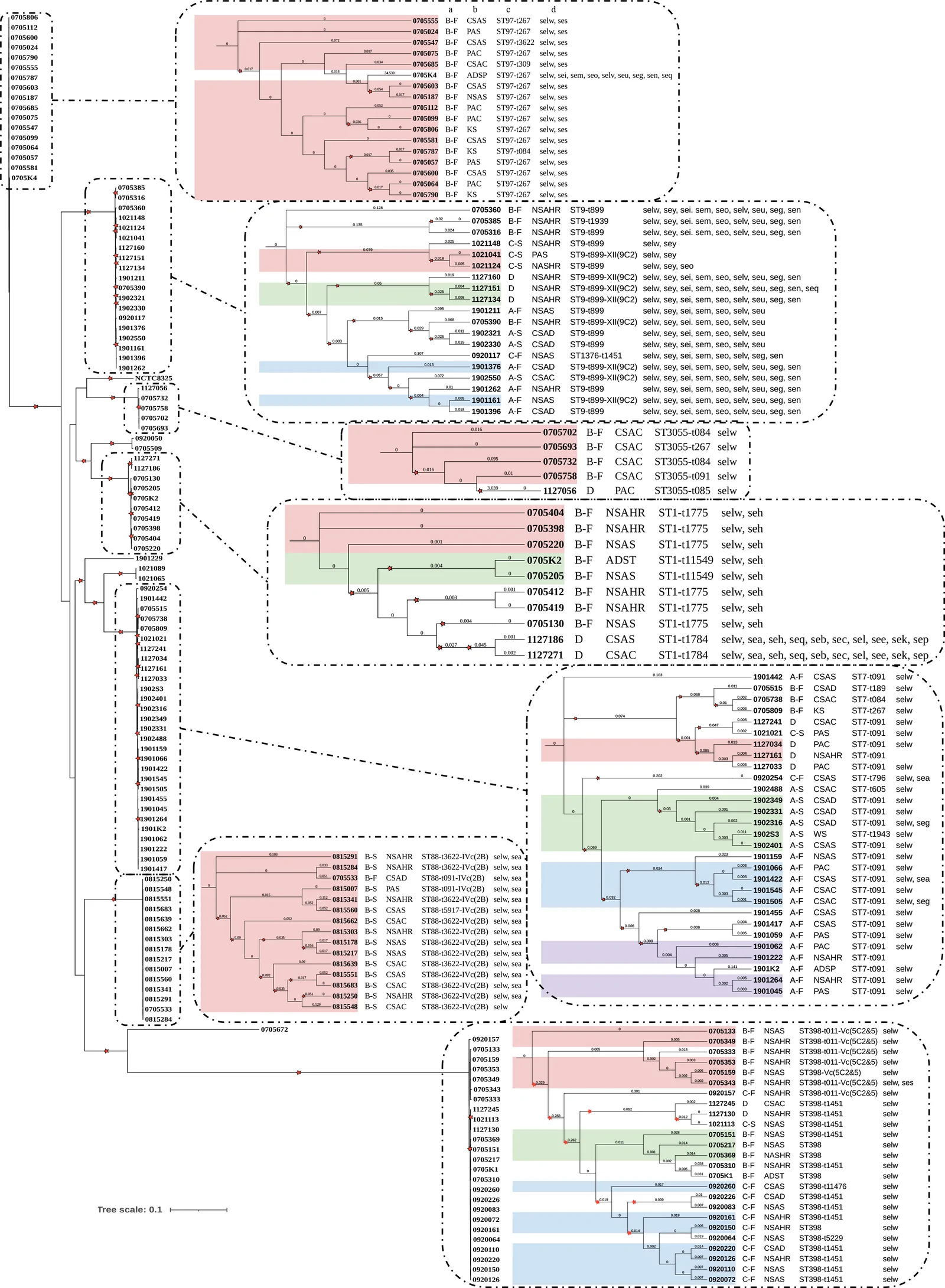
Phylogenetic evidence for abattoir transmission of 126 isolates. Maximum likelihood phylogenetic tree based on core genome SNPs of 126 isolates with 1,000 bootstraps, *S. aureus* NCTC8325 (txid: CP000253.1) as the reference genome, and the different phylogenetic groups were rebuilt, 0705130, 0920254, 0705385, 0815250, 0705806, 0705133, and 0705702 were used as reference genome for ST1, ST7, ST9, ST88, ST97, ST398, and ST3055, respectively. Branch lengths are shown above each branch, with a star representing 100% bootstrap support. Then, we assigned the isolates which shown the core-SNPs less than 30 to the same sublineage, after alignment in pairs. a: abattoirs (**A, B, C, and D**) and sampling visits (F: first visiting, S: second visiting); b: sample collections; c: genotypes of isolates; d: SE or SEl genes.

A comprehensive phylogenetic tree of 126 genomes was constructed based on the core-genome SNPs (Fig. 5). The results revealed that strains with the same ST were clustered in the same clade. To investigate transmission relationships, we reconstructed phylogenetic trees for each clade and assigned isolates with less than 30 core-SNPs to sublineages that may have undergone transmission events. The results showed one sublineage for ST97, ST3055, and ST88, two sublineages for ST1, three sublineages for ST9 and ST398, and four sublineages for ST7.

**Fig 6:**
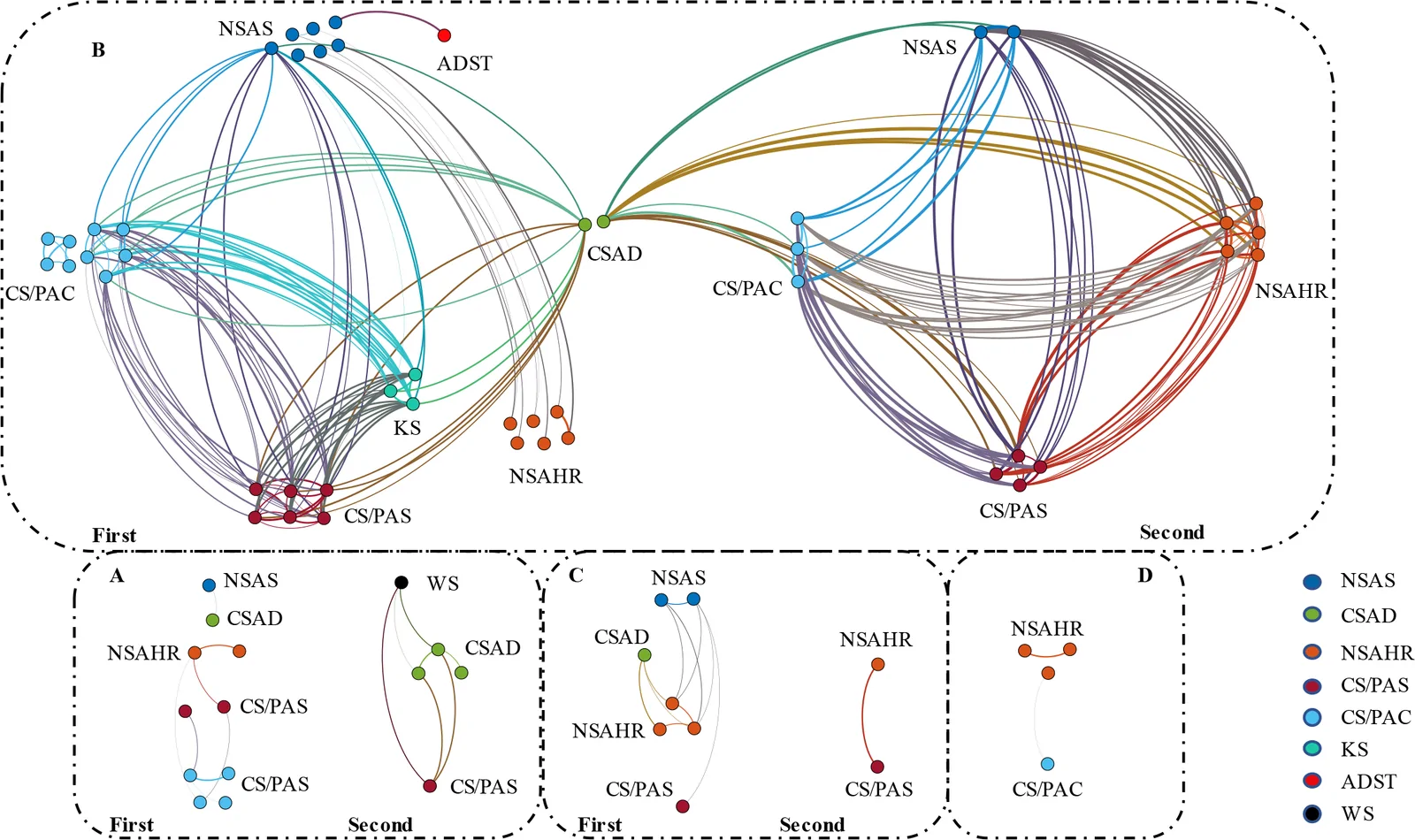
Association diagram showing the transmission of *S. aureus* based on the number of core-SNPs. Each dot represents a strain, colors correspond to the sample collections and the thickness of the connecting line corresponds to the differences. A, B, C and D: abattoirs in this study. First and second: different visiting. NSAS, nasal swabs after stunning; ADST, air deposition samples of stunning; CSAD, carcass swabs after dehairing; WS, water samples; NSAHR, nasal swabs after head-removal; CS/PAS, carcass swabs and pork after splitting; KS, knives swabs; CS/PAC, carcass swabs and pork after chilling.

Then, we constructed an association diagram to visually summarize transmission networks among different slaughtering stages in abattoirs (Fig. 6). Our results indicated that transmission occurred among all sample collections within each abattoir. For example, during the first visit to abattoir B, the transmission chain was ADST-NSAS, NSAS-NSAHR, and NSAS-CSAD-CS/PAS-KS-CS/PAC (first visit), and NSAS-NSAHR-CS/PAS-CS/PAC (second visit). Notably, a strain from CSAD during the first visit had extensive connections with isolates from the second visit. In abattoir A, the transmission chain was NSAS-CSAD, NSAHR-CS/PAS-CS/PAC (first visit), and WS-CSAD-CS/PAS (second visit). In abattoir C, the transmission chain was NSAS-CSAD-NSAHR-CS/PAS (first visit) and NSAHR-CS/PAS (second visit). In abattoir D, the transmission chain was NSAHR-CS/PAC. Together, these findings suggest that knives, water, and air are important factors in the transmission of enterotoxigenic *S. aureus*.

## DISCUSSION

**Fig 7:**
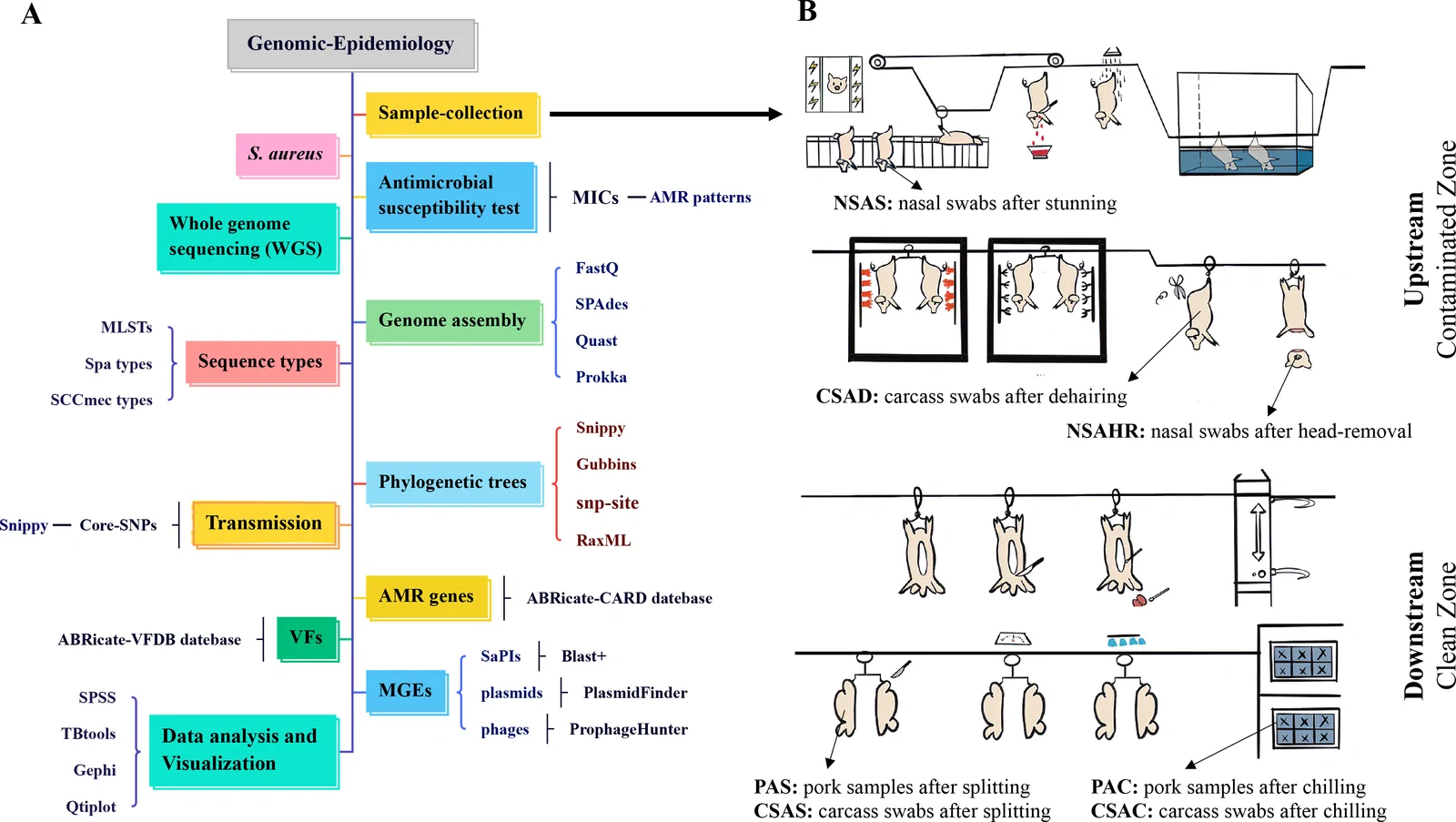
(**A**) The genomic-epidemiological research process used in this study. (**B**) Samples collection in abattoirs. Environment samples are not shown in figure. AMR, antimicrobial resistance; MGE, mobile genetic elements; MLSTs, multi-locus sequence types; SNPs, *single* nucleotide polymorphisms ; VFs, virulence factors.

The epidemiologic surveillance of pathogens can furnish substantial data for downstream decision-making and implementation of intervention measures. In this study, we proposed a genomic-epidemiology research process (Fig. 7). To our knowledge, this is the first time such a process is implemented to investigate the prevalence and transmission of *S. aureus* in abattoirs. It is noteworthy that, in addition to the conventional analysis process, we utilized core-SNPs (with a threshold set at 30) to determine sublineages, which provided more direct and compelling evidence for analyzing pathogens transmission.

The prevalence of *S. aureus* in abattoirs has been well documented (21, 22). Our study found an overall prevalence of 26.6% (966/3,638), similar to the prevalence (23.4%, 156/666) in pig farms reported in south China (23), but higher than that in European countries (22, 24). Among the different slaughter stages in abattoirs, our results showed that the lowest prevalence was samples of carcass swabs after dehairing (12.8%), indicating a significant reduction of pathogens attached to the carcass after the scalding pool. However, the high prevalence and close relationship between nasal swabs after stunning stage (33.4%) and head-removal stage (39.7%) suggested that the scalding pool had little effect on the carriage of *S. aureus* in the nasal cavity. During the splitting and chilling stages, we collected both pork samples and carcass swabs simultaneously. The prevalence of pork samples in the chilling stage was generally higher than that in the splitting stage, while the prevalence of carcass swab samples remained consistent. These results indicate that pork is more prone to bacterial accumulation through the entire slaughter process. Although disinfection measures such as rinsing are implemented between different stages, carcass can still be contaminated from the respective environments. The prevalence of *S. aureus* was basically the same at the beginning (stunning) and end (chilling) of the slaughter practice, but the sequence types were significantly different, demonstrating differences in contamination source. Based on the difference between clean and contaminated area, we divided the slaughter practice into upstream (from stunning to head-removal) and downstream (from splitting to refrigeration). The prevalence of upstream (29.0%) was significantly higher than that of downstream (24.4%). Most isolates of upstream belonged to ST9, ST88, and ST398, which are related to LA-MRSA (9, 25, 26), indicating that *S. aureus* from upstream mostly originated from pig farms. In contrast, ST7, ST97, and ST1 were more frequent sequence types in downstream, suggesting that they are more likely from the slaughter environment and workers (27 – 30). These findings suggested that the surveillance of *S. aureus* contamination in the farms may have limited risk assessment capabilities. After the slaughter and market stages, it becomes crucial to prioritize bacterial surveillance in pork at the consumer level.

Antimicrobial susceptibility testing revealed that *S. aureus* exhibited high proportions of resistance to antimicrobial agents, with 81.6% of isolates being MDR and all isolates showing resistance to at least one agent. Previous studies have reported similar high-level resistance among *S. aureus* (10, 31), highlighting an increasing prevalence of AMR or varied distribution across regions. In our study, all isolates were found to be resistant to SXT, while 84.4% and 79.9% of the isolates showed resistance to TET and ERY, respectively. Additionally, isolates recovered from upstream demonstrated significantly higher resistance proportions than those from downstream in CHL, CLI, ERY, and GEN, in this regard, *fexA*, *lsaE*, *ermC*, *ant(4')-Ib*, *aac(6')-Ie-APH(2'')-Ia*, and *ant (6)-Ia* might play critical roles (32 – 35). Although only a few ARGs were located on mobile elements, plasmids remain crucial in driving the transmission of ARGs (36, 37). We identified a total of 17 plasmid replicons mediating the transmission of ARGs (Fig. S4), including *tetK* and *tetL* for tetracycline resistance, *ermC* and *msrA* for macrolides resistance, *mecA* and *blaZ* for β-Lactam resistance, *ileS* for quinolones resistance, and *lunA* for lincomycin resistance.

Furthermore, a total of 159 VFs were identified. Our focus was on the enterotoxin genes (SE/SEl genes) of *S. aureus*, which are important superantigen toxins in food poisoning (2). A total of 19 different types of SE/SEl genes were identified, with *selw* (98.4%) having the highest carrying rate, followed by *sey* (15.1%), *sea* (14.3%), *sei* (14.3%), *sem* (14.3%), *seo* (14.3%), *ses* (14.3%), *selv* (14.3%), and *seg* (12.7%). We found a strong correlation between SE/SEl types and STs, such as ST9-*sei-seg-sem-sen-seo-seu-selv-selw-sey*, ST88*-sea-selw*, ST97*-ses-selw*, and ST1*-seh-selw*. The high prevalence of *selw* is consistent with previous studies (38, 39). SaPIs likely encode and disseminate SE/SEl genes (40). In this study, 10 SaPIs were identified, including SaPI363 and SaPI364P, which encode various SEs, while *seb* and *sec* are present in several SaPIs (Fig. S4). Additionally, we identified several virulence genes related to human pathogenesis, demonstrating considerable pathogenic potential for humans. These findings indicate that *S. aureus* harbors a wide range of ARGs and VFs, and MGEs mediating the transfer of various ARGs and VFs are prevalent. These factors contribute to increase of safety risks, difficulties in prevention and control of bacteria, as well as higher treatment costs.

After comparing and analyzing the core-SNPs, we established a 30-threshold to determine sublineage affiliation, followed by an investigation into their potential transmission routes (17). Our findings suggested that there was inter-sample transmission of *S. aureus* during slaughter practices, which can be attributed to the high density of slaughtering practices in abattoirs. Notably, we did not observe transmission of enterotoxigenic *S. aureus* between abattoirs, however, it was detected in both the first and second visits to abattoir B (Fig. 6), this stubborn bacterium may foster extensive contamination within abattoirs. Furthermore, knives, water, and air played critical roles in pathogen transmission along the contamination chain ADST-NSAS, NSAS-CSAD-CS/PAS-KS-CS/PAC, and WS-CSAD-CS/PAS, as previously reported (41 – 43). Therefore, reinforcing sterilization measures during slaughtering processes is of paramount importance in controlling the spread of enterotoxigenic *S. aureus*. It should be mentioned that our study only collected samples from abattoirs; thus, combining farm and market samples would provide more meaningful insights into the transmission chain of enterotoxigenic *S. aureus* from farm to table. However, given the complex breeding and market environments, the analysis methods and processes involved would be more intricate and challenging, and we are actively pursuing these avenues in future research.

Based on our research, the insights obtained are when developing surveillance plans for *S. aureus*, policymakers should prioritize post-production stages of the pork supply chain, specifically after the completion of the slaughter process, rather than solely concentrating on designing surveillance schemes at the farm level. To effectively control the transmission of *S. aureus* within slaughterhouses, it is crucial to implement measures that target equipment, water, and air as Hazard Analysis and Critical Control Points (HACCP) and improve disinfection protocols.

In conclusion, our study employed a genomic-epidemiological analysis process to demonstrate the prevalence, enterotoxigenic, and heterogeneity of enterotoxigenic *S. aureus* in pig abattoirs located in Hubei province. We also analyzed the significant difference of *S. aureus* recovered from upstream and downstream of the slaughter practice, and identified transmission relationship of enterotoxigenic *S. aureus* among different stages of slaughter. Furthermore, we confirmed the widespread presence of MDR *S. aureus* strains carrying various ARGs and virulence genes, as well as MGEs capable of mediating transmissibility. Therefore, it cannot be overemphasized to adopt a rational strategy for conducting continuous monitoring, implementing necessary interventions and control strategies of enterotoxigenic *S. aureus*.

## MATERIALS AND METHODS

### Genome-epidemiology research process, sample collection, and treatment

This study utilized a standardized genomic-epidemiological research process, as illustrated in Fig. 7A. Enterotoxigenic *S. aureus* was isolated and identified following sample collection. The isolates were subjected to antimicrobial susceptibility testing (based on sample collections) and WGS (based on the sample collections and AMR patterns), and sequence types and phylogenetic analyses were conducted to investigate the transmission. Furthermore, genomic characteristics were described by screening ARGs, virulence genes and MGEs. Finally, data analysis and visualization were performed to present the results.

Sampling was carried out seven visits in 2019 from four commercial pig abattoirs (designated as A, B, C, and D, two visits for each abattoir except abattoir D due to the COVID-19) located in Hubei province, according to the guideline of the Animal Management and Ethics Committee at Huazhong Agricultural University (the approval ID number: HZAUSW-2018–030). These abattoirs are part of large-scale pork processing enterprises, slaughtering average ~1,500–2,000 animals from intensive farms per day, and producing chilled pork for wholesale markets and supermarkets.

During each visit, samples were obtained from different batches and processing steps of animals and environment at each abattoir, as shown in Fig. 7B: NSAS, CSAD, NSAHR, CSAS, PAS, CSAC, PAC, and environmental samples (not shown in Fig. 7: air deposition samples, water samples , knife swabs , and ground swabs). Nasal swabs were collected by using sterile cotton buds premoistened with 0.1% buffered peptone water containing 10% of sodium chloride (BPW-10% NaCl). For carcass swabs, an area of approximately 100 cm^2^ was swabbed with sterile premoistened cotton buds. All swabs were aseptically placed into tubes containing 4 mL of BPW-10% NaCl, and then incubated for 18–24 hours at 37°C with shaking at 180–200 r/minute. Approximately 20 g of each pork sample, collected from the ventral rib area, was homogenized in 100 mL of BPW-10% NaCl and incubated for 18–24 hours at 37°C with shaking. Air deposition samples were collected by 50 mL tubes containing 40 mL of BPW-10% NaCl, and incubated for 18–24 hours at 37°C with shaking. Fifty milliliters of each flushing water sample was concentrated using sterilized 0.22 µm filter membranes, and then enriched in 2 mL of BPW-10% NaCl.

### Isolation and identification of *S. aureus*

Isolation of *S. aureus* was performed according to the Chinese National Standard GB4789 (http://down.foodmate.net/standard/sort/3/50371.html) with some modifications. The presumptive positive enrichment broth for *S. aureus* was confirmed after genomic DNA extraction and identification of *femB* gene by PCR (44), and streaked onto Baird-parker agar plates for incubation for 18–24 hours at 37°C. The single presumptive positive colony was picked, and the genomic DNA was extracted for PCR identification with *femB*. MRSA was confirmed based on the resistance to OXA and *mecA*/*mecC* positive (45, 46).

### Antimicrobial resistance testing

Antimicrobial resistance of *S. aureus* isolates was tested by determining the MICs using the broth microdilution method. Eight antimicrobial agents used in this study are as follows: CHL, CIP, CLI, ERY, OXA, GEN, TET, and SXT. The breakpoints set by the Clinical and Laboratory Standards Institute (47) were shown in Table S4. AMR was defined as resistance to at least one antimicrobial agent, whereas MDR was defined as resistance to three or more classes of antimicrobial agents. *S. aureus* ATCC29213 was used as the quality control strain.

### Whole-genome sequencing and bioinformatics analysis

The genomic DNA was extracted using the E.Z.N.A. bacteria DNA Extraction Kit (Omega Bio-tek. Inc. Georgia, USA). Library preparation and Denovo Bacterial Sequencing were conducted on the Illumina platforms with NovaSeq 6000 Sequencing System at Annoroad Gene Technology Co., Ltd, Beijing. All subsequent steps were performed on the instrument, including cluster generation and 2 × 150 paired-end sequencing. Genome assembly was conducted using SPAdes ver. 3.14.1 (48), using clean data after quantity control and data filter by FastQ-tools (https://github.com/dcjones/fastq-tools), with the “careful” option and the default K-mers (33, 55, and 77). Quast ver. 5.0.2 (49) was used to evaluate the quantity of the assembled contigs. Prokka (50) was used for gene prediction and genome annotation. Resistance genes and virulence genes were screened using ABRicate ver. 1.0.1 (https://github.com/tseemann/abricate) with the CARD (51) and VFDB (52) databases, respectively. PlasmidFinder (53) and Prophage Hunter (54) were used to screen plasmids and prophages, respectively. SaPIs screening were performed using Blast+ ver. 2.13.0 (55), and SaPIs sequences were collected from the NCBI nucleic-acid database (Table S4). Heatmaps of these sequences were created using TBtools (56).

### Transmission of *S. aureus* based on the core-SNPs

To investigate the transmission of *S. aureus* among abattoirs, we initiated by identifying the core-genome using Snippy ver. 3.2 (http://github.com/tseemann/snippy), followed by detecting and removal of recombination sequences using Gubbins (57), then the core-SNPs were extracted using snp-sites (58). The maximum likelihood tree of core-SNPs was constructed by RaxML (59) with 1000 bootstraps; the reference genome being *S. aureus* NCTC8325 (txid: CP000253.1). Based on the phylogenetic tree, we performed paired comparisons of core-SNPs for all isolates, which led to the identification of strains with core-SNPs of ≤30 (17), and subsequently grouped them into a sublineage. If isolates from various sample collections belonged to the same sublineage, it suggested potential transmission.

### Statistical analysis

Data analyses were performed using SPSS software ver. 26.0 (SPSS inc., Chicago, IL, USA). The confidence intervals were calculated using the approach outlined by Ross (60). Regression logistics analysis was carried out to examine the variation between sample collections (Table S1). The statistical significance between percentages of different groups was compared using the chi-squared test, with a *P*-value < 0.05 being considered significant.

## Data Availability

The genomic data produced in this study are available under BioProject accession PRJNA884517 and BioSamples SAMN31029732-SAMN31029857.

## SUPPLEMENTAL MATERIAL

Supplemental material spectrum.01913-23-s0001.pdf Tables S1 to S6; Fig. S1 to S4.

ASM does not own the copyrights to Supplemental Material that may be linked to, or accessed through, an article. The authors have granted ASM a non-exclusive, world-wide license to publish the Supplemental Material files. Please contact the corresponding author directly for reuse.
