# Supplementary material for: Heterogeneity and transmission of food safety-related enterotoxigenic Staphylococcus aureus in pig abattoirs in Hubei, China
Source: Microbiol Spectr. 2023 Sep 29;11(5):e01913-23. doi: 10.1128/spectrum.01913-23 (PMC10581196; doi:10.1128/spectrum.01913-23)
Supplement: Supplemental material — Tables S1 to S6; Fig. S1 to S4. [file spectrum.01913-23-s0001.pdf]

Table S1. Logistic Regression Analysis of the Presence of *S.aureus* in Different Factors.

Factors	Comparison	B *Regression Coefficient*	*SE*	p	*OR*	*95% CI*	
						Lower	*Upper*
Chain							
	Upstream vs. Downstream	0.236	0.075	0.002	1.266	1.092	1.467
Abattoirs							
	A vs. B	0.168	0.089	0.061	1.183	0.992	1.409
	A vs. C	0.304	0.110	0.006	1.355	1.092	1.683
	A vs. D	-0.360	0.144	0.013	0.697	0.525	0.925
	B vs. C	0.136	0.105	0.193	1.146	0.933	1.407
	B vs. D	-0.528	0.140	0.000	0.590	0.448	0.776
	C vs. D	-0.664	0.154	0.000	0.515	0.380	0.696
Sample Collections							
	NSAS vs. NSAHR	-0.274	0.124	0.027	0.761	0.597	0.969
	NSAS vs. CSAD	1.109	0.159	0.000	3.030	2.220	4.136
	NSAS vs. CSAS	0.516	0.133	0.000	1.676	1.293	2.173
	NSAS vs. CSAC	0.525	0.133	0.000	1.691	1.302	2.195
	NSAS vs. PAS	0.699	0.160	0.000	2.013	1.470	2.755
	NSAS vs. PAC	-0.024	0.169	0.886	0.976	0.701	1.359
	NSAHR vs. CSAD	1.504	0.154	0.000	4.500	3.331	6.081
	NSAHR vs. CSAS	0.790	0.127	0.000	2.204	1.717	2.829
	NSAHR vs. CSAC	0.799	0.128	0.000	2.223	1.729	2.858
	NSAHR vs. PAS	0.973	0.156	0.000	2.646	1.949	3.592
	NSAHR vs. PAC	0.250	0.165	0.130	1.283	0.929	1.773
	CSAD vs. CSAS	-0.714	0.161	0.000	0.490	0.357	0.671
	CSAD vs. CSAC	-0.705	0.161	0.000	0.494	0.360	0.678
	CSAD vs. PAS	-0.531	0.184	0.004	0.588	0.410	0.844
	CSAD vs. PAC	-1.255	0.192	0.000	0.285	0.196	0.415
	CSAS vs. CSAC	0.009	0.137	0.949	1.009	0.771	1.319
	CSAS vs. PAS	0.183	0.163	0.262	1.201	0.872	1.653
	CSAS vs. PAC	-0.541	0.172	0.002	0.582	0.416	0.815
	CSAC vs. PAS	0.174	0.164	0.287	1.190	0.864	1.641
	CSAC vs. PAC	-0.549	0.172	0.001	0.577	0.412	0.809
	PAS vs. PAC	-0.724	0.194	0.000	0.485	0.332	0.709

SE: standard error; *OR*: odds ratio; NSAS: nasal swabs after stunning; CSAD: carcass swabs after dehairing; NSAHR: nasal swabs after head-removal; CSAS: carcass swabs after splitting; PAS: pork after splitting; CSAC: carcass swabs after chilling; PAC: pork after chilling.

Table S2. AMR patterns of 543 *S. aureus*.

AMR patterns	Upstream			Downstream			Total
	No. of isolates	proportion (%)	95% CI	No. of isolates	proportion (%)	95% CI	
ERY-SUL-TET	16	5.69	3.3-9.1	51	19.47	14.9-24.8	67
CHL-CIP-CLI-ERY-GEN-SUL-TET	57	20.28	15.7-25.5	1	0.38	0.0-2.1	58
CHL-CIP-CLI-ERY-SUL-TET	46	16.37	12.2-21.2	7	2.67	1.1-5.4	53
SUL-TET	10	3.56	1.7-6.4	37	14.12	10.1-18.9	47
CLI-ERY-OXA-SUL-TET	2	0.71	0.1-2.5	36	13.74	9.8-18.5	38
CLI-ERY-SUL-TET	15	5.34	3.0-8.7	15	5.73	3.2-9.3	30
ERY-SUL	16	5.69	3.3-9.1	14	5.34	3.0-8.8	30
CHL-CIP-CLI-ERY-GEN-OXA-SUL-TET	21	7.47	4.7-11.2	5	1.91	0.6-4.4	26
SUL	7	2.49	1.0-5.1	15	5.73	3.2-9.3	22
CHL-CLI-ERY-SUL-TET	15	5.34	3.0-8.7	6	2.29	0.8-4.9	21
CHL-CLI-ERY-OXA-SUL-TET	8	2.85	1.2-5.5	7	2.67	1.1-5.4	15
CIP-CLI-ERY-GEN-SUL-TET	15	5.34	3.0-8.7				15
CHL-ERY-SUL-TET	3	1.07	0.2-3.1	11	4.20	2.1-7.4	14
CLI-OXA-SUL-TET	9	3.20	1.5-6.0	5	1.91	0.6-4.4	14
CLI-OXA-SUL				13	4.96	2.7-8.3	13
CLI-ERY-OXA-SUL	2	0.71	0.1-2.5	9	3.44	1.6-6.4	11
CLI-ERY-GEN-SUL-TET	3	1.07	0.2-3.1	6	2.29	0.8-4.9	9
CHL-CLI-ERY-GEN-SUL-TET	5	1.78	0.6-4.1	1	0.38	0.0-2.1	6
CLI-ERY-GEN-OXA-SUL-TET	2	0.71	0.1-2.5	4	1.53	0.4-3.9	6
CHL-CLI-ERY-GEN-SUL	6	2.14	0.8-4.6				6
CHL-ERY-OXA-SUL-TET				5	1.91	0.6-4.4	5
CIP-CLI-ERY-SUL-TET	5	1.78	0.6-4.1				5
CHL-CLI-ERY-GEN-OXA-SUL-TET				4	1.53	0.4-3.9	4
CHL-CIP-CLI-GEN-OXA-SUL-TET	3	1.07	0.2-3.1				3
CHL-CIP-ERY-SUL-TET	3	1.07	0.2-3.1				3
CHL-CIP-SUL-TET	3	1.07	0.2-3.1				3
ERY-GEN-SUL-TET	2	0.71	0.1-2.5	1	0.38	0.0-2.1	3
CIP-CLI-ERY-GEN-OXA-SUL-TET	2	0.71	0.1-2.5				2
CLI-ERY-GEN-SUL				2	0.76	0.1-2.7	2
CLI-GEN-OXA-SUL-TET				1	0.38	0.0-2.1	1
CHL-CLI-SUL-TET	1	0.36	0.0-0.2				1
CHL-GEN-SUL-TET	1	0.36	0.0-0.2				1
CIP-OXA-SUL-TET	1	0.36	0.0-0.2				1
CLI-ERY-GRN-SUL				1	0.38	0.0-2.1	1
CLI-GEN-SUL-TET				1	0.38	0.0-2.1	1
ERY-OXA-SUL-TET				1	0.38	0.0-2.1	1
CHL-SUL-TET	1	0.36	0.0-0.2				1
CLI-ERY-SUL				1	0.38	0.0-2.1	1
CLI-SUL-TET				1	0.38	0.0-2.1	1
ERY-OXA-SUL				1	0.38	0.0-2.1	1
CIP-SUL	1	0.36	0.0-0.2				1
Total	281			262			543

CI: confidence intervals.

Table S3. Distribution of 126 *S. aureus* according to different sample collections.

Clonal Complex	Sequence Type	Spa Type	SCCmec Type	Upstream					Up Total	Downstream				Down Total
				NSAS	ADST	CSAD	WS	NSAHR		CS/PAS	ADSP	KS	CS/PAC	
CC1	ST1	t1775		2				4	6					
		t1784								1			1	2
		t11549		1	1				2					
	ST9	t899				2		2	4					
			XII(9C2)	1		1		5	7	1			1	2
			-	1		1		2	4					
		t1939						1	1					
	ST188	t693				1			1					
		t5917								1				1
	ST1376			1					1					
CC5	ST6	-		2					2					
CC15	ST3055	t084											2	2
		t085											1	1
		t091											1	1
		t267											1	1
CC22	ST22												1	1
CC97	ST97	t084										1		1
		t267		1					1	6	1	2	5	14
		t3622								1				1
-	ST7	t084											1	1
		t091		1		3		3	7	8	1		7	16
		t189				1			1					
		t267										1		1
		t605											1	1
		t796								1				1
		t1943					1		1					
-	ST398	t011	Vc(5C2&5)	1				5	6					
		t1451		5		2		4	11				1	1
		t5229		1					1					
		t11476								1				1
		-	Vc(5C2&5)	1					1					
				1	1			2	4					
-	ST88	t091	IVc(2B)			1			1	1				1
		t3622	IVc(2B)	2				5	7	2			3	5
		t5917	IVc(2B)							1				1
-	ST2867							1	1					
Total				21	2	12	1	34	70	24	2	4	26	56

"-", Indicates that there is no corresponding types. NSAS: nasal swabs after stunning; ADST: air deposition samples of stunning; CSAD: carcass swabs after dehairing; WS: water samples; NSAHR: nasal swabs after head-removal; CS/PAS: carcass swabs and pork after splitting; ADSP: air deposition samples of splitting; KS: knives swabs; CS/PAC: carcass swabs and pork after chilling.

Table S4. MIC breakpoints in *S. aureus* in this study.

Antibiotics	MIC breakpoints (ug/ml)		
	S	I	R
Chloramphenicol (CHL)	≤8	16	≥32
Ciprofloxacin (CIP)	≤1	2	≥4
Clindamycin (CLI)	≤0.5	1~2	≥4
Erythromycin (ERY)	≤0.5	1~4	≥8
Oxacillin (OXA)	≤2	-	≥4
Gentamycin (GEN)	≤4	8	≥16
Tetracycline (TET)	≤4	8	≥16
Sulfamethoxazole-Trimethoprim (SXT)	≤2/38	-	≥4/76

"-", No corresponding criterion; R: resistant; I: intermediate; S: susceptible. The breakpoints were setting by the Clinical and Laboratory Standards Institute (M100: ISBN 978-1-68440-033-1).

Table S5. Accession numbers of SaPIs used in this study.

SaPI	Accession number	SaPI	Accession number
SaPI1	AB983198.1	SaPIhhms2	AB704540.1
SaPI2	EF010993.1	SaPIHirosaki4	AB716352.1
SaPI2R	AB983196.1	SaPIIshikawa11	AB716350.1
SaPI3	AF410775.1	SaPIIVM10	AB716349.1
SaPI356P	MN450305.1	SaPIivm60	AB704539.1
SaPI363P	MN450303.1	SaPIj11	AB704541.1
SaPI364P	MN450304.1	SaPIj50	LC606231.1
SaPI68111	JN689383.1	SaPIJICS137	LC517303.1
SaPIABD2001	MH823211.1	SaPINN54	AB690437.1
SaPIbov	AF217235.1	SaPINO.10	AB716351.1
SaPIbov2	AY220730.1	SaPIPM1	AB690438.1
SaPIbov4	HM211303.1	SaPITokyo11212	AB860416.1
SaPIbov5	HM228919.1	SaPITokyo12381	AB860418.1
SaPIeq1	HM228920.1	SaPITokyo12413	AB860415.1
SaPIfhuD	AB983199.1	SaPITokyo12571	AB860417.1

Table S6. Information of 126 S. aureus isolates and antimicrobial resistance phenotypes.

Biosamples	Strains	Sampling date	Sampling location	Sampling collections	Farm location	Species	MSSA/ MRSA	MLST	Spa types	SCCmec types
SAMN31029732	0705024	2019.7.05	B	PAS	HCWS	S.aureus	MSSA	97	t267	
SAMN31029733	0705057	2019.7.05	B	PAS	HAZB	S.aureus	MSSA	97	t267	
SAMN31029734	0705064	2019.7.05	B	PAC	HCWS	S.aureus	MSSA	97	t267	
SAMN31029735	0705075	2019.7.05	B	PAC	HCWS	S.aureus	MSSA	97	t267	
SAMN31029736	0705099	2019.7.05	B	PAC	HAZB	S.aureus	MSSA	97	t267	
SAMN31029737	0705112	2019.7.05	B	PAC	HAZB	S.aureus	MSSA	97	t267	
SAMN31029738	0705130	2019.7.05	B	NSAS	HCWS	S.aureus	MSSA	1	t1775	
SAMN31029739	0705133	2019.7.05	B	NSAS	HCWS	S.aureus	MRSA	398	t011	Vc(5C2&5)
SAMN31029740	0705151	2019.7.05	B	NSAS	HAZB	S.aureus	MSSA	398	t1451	
SAMN31029741	0705159	2019.7.05	B	NSAS	HAZB	S.aureus	MRSA	398	/	Vc(5C2&5)
SAMN31029742	0705187	2019.7.05	B	NSAS	HCWS	S.aureus	MSSA	97	t267	
SAMN31029743	0705205	2019.7.05	B	NSAS	HCWS	S.aureus	MSSA	1	t11549	
SAMN31029744	0705217	2019.7.05	B	NSAS	HCWS	S.aureus	MSSA	398	/	
SAMN31029745	0705220	2019.7.05	B	NSAS	HCWS	S.aureus	MSSA	1	t1775	
SAMN31029746	0705310	2019.7.05	B	NSAHR	HCWS	S.aureus	MSSA	398	t1451	
SAMN31029747	0705316	2019.7.05	B	NSAHR	HCWS	S.aureus	MSSA	9	t899	
SAMN31029748	0705333	2019.7.05	B	NSAHR	HAZB	S.aureus	MRSA	398	t011	Vc(5C2&5)
SAMN31029749	0705343	2019.7.05	B	NSAHR	HAZB	S.aureus	MRSA	398	t011	Vc(5C2&5)
SAMN31029750	0705349	2019.7.05	B	NSAHR	HAZB	S.aureus	MRSA	398	t011	Vc(5C2&5)
SAMN31029751	0705353	2019.7.05	B	NSAHR	HAZB	S.aureus	MRSA	398	t011	Vc(5C2&5)
SAMN31029752	0705360	2019.7.05	B	NSAHR	HAZB	S.aureus	MRSA	9	t899	-
SAMN31029753	0705369	2019.7.05	B	NSAHR	HCWS	S.aureus	MSSA	398	/	
SAMN31029754	0705385	2019.7.05	B	NSAHR	HCWS	S.aureus	MSSA	9	t1939	
SAMN31029755	0705390	2019.7.05	B	NSAHR	HCWS	S.aureus	MRSA	9	t899	XII(9C2)
SAMN31029756	0705398	2019.7.05	B	NSAHR	HCWS	S.aureus	MSSA	1	t1775	
SAMN31029757	0705404	2019.7.05	B	NSAHR	HCWS	S.aureus	MSSA	1	t1775	
SAMN31029758	0705412	2019.7.05	B	NSAHR	HCWS	S.aureus	MSSA	1	t1775	
SAMN31029759	0705419	2019.7.05	B	NSAHR	HCWS	S.aureus	MSSA	1	t1775	
SAMN31029760	0705509	2019.7.05	B	CSAD	HCWS	S.aureus	MSSA	1	t693	
SAMN31029761	0705515	2019.7.05	B	CSAD	HCWS	S.aureus	MSSA	7	t189	
SAMN31029762	0705533	2019.7.05	B	CSAD	HCWS	S.aureus	MRSA	88	t091	IVc(2B)
SAMN31029763	0705547	2019.7.05	B	CSAS	HCWS	S.aureus	MSSA	97	t3622	
SAMN31029764	0705555	2019.7.05	B	CSAS	HCWS	S.aureus	MSSA	97	t267	
SAMN31029765	0705581	2019.7.05	B	CSAS	HAZB	S.aureus	MSSA	97	t267	
SAMN31029766	0705600	2019.7.05	B	CSAS	HAZB	S.aureus	MSSA	97	t267	
SAMN31029767	0705603	2019.7.05	B	CSAS	HCWS	S.aureus	MSSA	97	t267	
SAMN31029768	0705672	2019.7.05	B	CSAC	HCWS	S.aureus	MSSA	22	t267	
SAMN31029769	0705685	2019.7.05	B	CSAC	HCWS	S.aureus	MSSA	97	t309	
SAMN31029770	0705693	2019.7.05	B	CSAC	HAZB	S.aureus	MSSA	3055	t267	
SAMN31029771	0705702	2019.7.06	B	CSAC	HAZB	S.aureus	MSSA	3055	t084	
SAMN31029772	0705732	2019.7.05	B	CSAC	HCWS	S.aureus	MSSA	3055	t084	
SAMN31029773	0705738	2019.7.05	B	CSAC	HCWS	S.aureus	MSSA	7	t084	
SAMN31029774	0705758	2019.7.05	B	CSAC	HCWS	S.aureus	MSSA	3055	t091	
SAMN31029775	0705787	2019.7.05	B	KSP	WHSH	S.aureus	MSSA	97	t084	
SAMN31029776	0705790	2019.7.05	B	KSP	WHSH	S.aureus	MSSA	97	t267	
SAMN31029777	0705806	2019.7.05	B	KSP	WHSH	S.aureus	MSSA	97	t267	

SAMN31029778	0705809	2019.7.05	B	KSP	WHSH	S.aureus	MSSA	7	t267	
SAMN31029779	0815007	2019.8.15	B	PAS	XC	S.aureus	MRSA	88	t091	IVc(2B)
SAMN31029780	0815178	2019.8.15	B	NSAS	XGHC	S.aureus	MRSA	88	t3622	IVc(2B)
SAMN31029781	0815217	2019.8.15	B	NSAS	XNXA	S.aureus	MRSA	88	t3622	IVc(2B)
SAMN31029782	0815250	2019.8.15	B	NSAHR	XC	S.aureus	MRSA	88	t3622	IVc(2B)
SAMN31029783	0815284	2019.8.15	B	NSAHR	XGHC	S.aureus	MRSA	88	t3622	IVc(2B)
SAMN31029784	0815291	2019.8.15	B	NSAHR	XGHC	S.aureus	MRSA	88	t3622	IVc(2B)
SAMN31029785	0815303	2019.8.15	B	NSAHR	HSDY	S.aureus	MRSA	88	t3622	IVc(2B)
SAMN31029786	0815341	2019.8.15	B	NSAHR	XNXA	S.aureus	MRSA	88	t3622	IVc(2B)
SAMN31029787	0815548	2019.8.15	B	CSAS	HSDY	S.aureus	MRSA	88	t3622	IVc(2B)
SAMN31029788	0815551	2019.8.15	B	CSAS	HSDY	S.aureus	MRSA	88	t3622	IVc(2B)
SAMN31029789	0815560	2019.8.15	B	CSAS	HSDY	S.aureus	MRSA	88	t5917	IVc(2B)
SAMN31029790	0815639	2019.8.15	B	CSAC	XGHC	S.aureus	MRSA	88	t3622	IVc(2B)
SAMN31029791	0815662	2019.8.15	B	CSAC	HSDY	S.aureus	MRSA	88	t3622	IVc(2B)
SAMN31029792	0815683	2019.8.15	B	CSAC	HSDY	S.aureus	MRSA	88	t3622	IVc(2B)
SAMN31029793	0920050	2019.9.20	C	PAS	CH-1	S.aureus	MSSA	188	t5917	
SAMN31029794	0920064	2019.9.20	C	NSAS	GC	S.aureus	MSSA	398	t5229	
SAMN31029795	0920072	2019.9.20	C	NSAS	GC	S.aureus	MSSA	398	t1451	
SAMN31029796	0920083	2019.9.20	C	NSAS	GC	S.aureus	MSSA	398	t1451	
SAMN31029797	0920110	2019.9.20	C	NSAS	CH-1	S.aureus	MSSA	398	t1451	
SAMN31029798	0920117	2019.9.20	C	NSAS	CH-1	S.aureus	MSSA	1376	t1451	
SAMN31029799	0920126	2019.9.20	C	NSAHR	GC	S.aureus	MSSA	398	t1451	
SAMN31029800	0920150	2019.9.20	C	NSAHR	GC	S.aureus	MSSA	398	/	
SAMN31029801	0920157	2019.9.20	C	NSAHR	CH-1	S.aureus	MRSA	398	t011	Vc(5C2&5)
SAMN31029802	0920161	2019.9.20	C	NSAHR	CH-1	S.aureus	MSSA	398	t1451	
SAMN31029803	0920220	2019.9.20	C	CSAD	CH-1	S.aureus	MSSA	398	t1451	
SAMN31029804	0920226	2019.9.20	C	CSAD	CH-1	S.aureus	MSSA	398	t1451	
SAMN31029805	0920254	2019.9.20	C	CSAS	GC	S.aureus	MSSA	7	t796	
SAMN31029806	0920260	2019.9.20	C	CSAS	GC	S.aureus	MSSA	398	t11476	
SAMN31029807	1021021	2019.10.21	C	PAS	CH-2	S.aureus	MSSA	7	t091	
SAMN31029808	1021041	2019.10.21	C	PAS	WJ	S.aureus	MRSA	9	t899	XII(9C2)
SAMN31029809	1021065	2019.10.21	C	NSAS	CH-2	S.aureus	MSSA	9	/	
SAMN31029810	1021089	2019.10.21	C	NSAS	CH-2	S.aureus	MSSA	6	/	
SAMN31029811	1021113	2019.10.21	C	NSAS	WJ	S.aureus	MSSA	398	t1451	
SAMN31029812	1021124	2019.10.21	C	NSAHR	CH-2	S.aureus	MSSA	9	t899	
SAMN31029813	1021148	2019.10.21	C	NSAHR	CH-2	S.aureus	MRSA	398	t899	XII(9C2)
SAMN31029814	1127033	2019.11.28	D	PAC	MYA	S.aureus	MSSA	7	t091	
SAMN31029815	1127034	2019.11.28	D	PAC	MYA	S.aureus	MSSA	7	t091	
SAMN31029816	1127056	2019.11.28	D	PAC	MYB	S.aureus	MSSA	3055	t085	
SAMN31029817	1127130	2019.11.28	D	NSAHR	MYA	S.aureus	MSSA	9	t1451	
SAMN31029818	1127134	2019.11.28	D	NSAHR	MYA	S.aureus	MRSA	7	t899	XII(9C2)
SAMN31029819	1127151	2019.11.28	D	NSAHR	MYB	S.aureus	MRSA	1	t899	XII(9C2)
SAMN31029820	1127160	2019.11.28	D	NSAHR	MYB	S.aureus	MRSA	7	t899	XII(9C2)
SAMN31029821	1127161	2019.11.28	D	NSAHR	MYB	S.aureus	MSSA	1	t091	
SAMN31029822	1127186	2019.11.28	D	CSAS	MYA	S.aureus	MSSA	398	t1784	
SAMN31029823	1127241	2019.11.28	D	CSAC	MYA	S.aureus	MSSA	9	t091	
SAMN31029824	1127245	2019.11.28	D	CSAC	MYA	S.aureus	MSSA	9	t1451	
SAMN31029825	1127271	2019.11.28	D	CSAC	MYB	S.aureus	MSSA	398	t1784	

SAMN31029826	1901045	2019.3.15	A	PAS	SP	S.aureus	MSSA	7	t091	
SAMN31029827	1901059	2019.3.15	A	PAS	SP	S.aureus	MSSA	7	t091	
SAMN31029828	1901062	2019.3.15	A	PAC	SP	S.aureus	MSSA	7	t091	
SAMN31029829	1901066	2019.3.15	A	PAC	SP	S.aureus	MSSA	7	t091	
SAMN31029830	1901159	2019.3.15	A	NSAS	SP	S.aureus	MSSA	7	t091	
SAMN31029831	1901161	2019.3.15	A	NSAS	SP	S.aureus	MRSA	9	t899	XII(9C2)
SAMN31029832	1901211	2019.3.15	A	NSAS	GS	S.aureus	MRSA	9	t899	-
SAMN31029833	1901222	2019.3.15	A	NSAHR	HP	S.aureus	MSSA	7	t091	
SAMN31029834	1901229	2019.3.15	A	NSAHR	HP	S.aureus	MSSA	2867	t2016	
SAMN31029835	1901262	2019.3.15	A	NSAHR	SP	S.aureus	MRSA	9	t899	-
SAMN31029836	1901264	2019.3.15	A	NSAHR	SP	S.aureus	MSSA	7	t091	
SAMN31029837	1901376	2019.3.15	A	CSAD	GS	S.aureus	MRSA	9	t899	XII(9C2)
SAMN31029838	1901396	2019.3.15	A	CSAD	GS	S.aureus	MRSA	9	t899	-
SAMN31029839	1901417	2019.3.15	A	CSAS	HP	S.aureus	MSSA	7	t091	
SAMN31029840	1901422	2019.3.15	A	CSAS	HP	S.aureus	MSSA	7	t091	
SAMN31029841	1901442	2019.3.15	A	CSAS	SP	S.aureus	MSSA	7	t091	
SAMN31029842	1901455	2019.3.15	A	CSAS	SP	S.aureus	MSSA	7	t091	
SAMN31029843	1901505	2019.3.15	A	CSAC	HP	S.aureus	MSSA	7	t091	
SAMN31029844	1901545	2019.3.15	A	CSAC	SP	S.aureus	MSSA	7	t091	
SAMN31029845	1902316	2019.4.18	A	CSAD	JK	S.aureus	MSSA	7	t091	
SAMN31029846	1902321	2019.4.18	A	CSAD	JK	S.aureus	MSSA	9	t091	
SAMN31029847	1902330	2019.4.18	A	CSAD	JK	S.aureus	MSSA	9	t899	
SAMN31029848	1902331	2019.4.18	A	CSAD	SP	S.aureus	MSSA	7	t899	
SAMN31029849	1902349	2019.4.18	A	CSAD	SP	S.aureus	MSSA	7	t091	
SAMN31029850	1902401	2019.4.18	A	CSAS	JK	S.aureus	MSSA	7	t091	
SAMN31029851	1902488	2019.4.18	A	CSAC	JK	S.aureus	MSSA	7	t605	
SAMN31029852	1902550	2019.4.18	A	CSAC	GS	S.aureus	MRSA	9	t899	XII(9C2)
SAMN31029853	0705K1	2019.7.05	B	AWL	WHSH	S.aureus	MSSA	398	/	
SAMN31029854	0705K2	2019.7.05	B	AAST	WHSH	S.aureus	MSSA	1	t11549	
SAMN31029855	0705K4	2019.7.05	B	AASP	WHSH	S.aureus	MSSA	97	t267	
SAMN31029856	1901K2	2019.3.15	A	AASP	WHZL	S.aureus	MSSA	7	t091	
SAMN31029857	1902S3	2019.4.18	A	WAD	WHZL	S.aureus	MSSA	7	t1943	

AMR patterns	CHL	CIP	CLI	ERY	OXA	GEN	TET	SUL
SUL-TET	I	S	S	S	S	S	R	R
SUL-TET	I	S	S	S	S	S	R	R
SUL-TET	I	S	S	S	S	S	R	R
CHL-CIP-CLI-ERY-GEN-SUL-TET	R	R	R	R	R	S	R	R
SUL-TET	I	S	S	S	S	S	R	R
SUL-TET	I	S	S	S	S	S	R	R
CHL-CLI-ERY-GEN-OXA-SUL-TET	R	S	R	R	R	R	R	R
CLI-ERY-OXA-SUL-TET	I	S	R	R	S	R	R	R
CHL-CIP-CLI-ERY-GEN-SUL-TET	R	R	R	R	R	S	R	R
CLI-ERY-OXA-SUL-TET	S	S	R	R	S	R	R	R
CLI-CIP-ERY-SUL-TET	I	R	R	R	S	S	R	R
CHL-CIP-CLI-ERY-SUL-TET	R	R	R	R	I	S	R	R
CHL-CIP-CLI-ERY-GEN-SUL-TET	R	R	R	R	R	S	R	R
CHL-CLI-ERY-GEN-SUL-TET	R	S	R	R	R	S	R	R
CHL-CIP-CLI-ERY-SUL-TET	R	R	R	R	I	S	R	R
CIP-CLI-ERY-GEN-SUL-TET	I	R	R	R	R	S	R	R
CLI-ERY-OXA-SUL-TET	S	S	R	R	S	R	R	R
CLI-ERY-OXA-SUL-TET	I	S	R	R	S	R	R	R
CLI-OXA-SUL-TET	S	S	R	S	S	R	R	R
CLI-ERY-OXA-SUL-TET	I	S	R	R	S	R	R	R
CHL-CIP-CLI-ERY-GEN-OXA-SUL-TET	R	R	R	R	R	R	R	R
CHL-CIP-CLI-ERY-GEN-SUL-TET	R	R	R	R	R	S	R	R
CHL-CIP-CLI-ERY-GEN-SUL-TET	R	R	R	R	R	S	R	R
CHL-CIP-CLI-ERY-GEN-OXA-SUL-TET	R	R	R	R	R	R	R	R
CHL-CLI-ERY-GEN-SUL-TET	R	S	R	R	R	S	R	R
CHL-CLI-ERY-GEN-SUL-TET	R	S	R	R	R	S	R	R
CHL-CLI-ERY-GEN-SUL-TET	R	S	R	R	R	S	R	R
CHL-CLI-ERY-GEN-SUL-TET	R	S	R	R	R	S	R	R
CHL-ERY-GEN-SUL-TET	R	S	I	R	R	S	R	R
CLI-ERY-GEN-SUL-TET	I	S	R	R	R	S	R	R
ERY-OXA-SUL	S	S	I	R	S	R	R	S
SUL-TET	S	S	S	S	S	S	R	R
SUL-TET	S	S	S	S	S	S	R	R
SUL-TET	S	S	S	S	S	S	R	R
SUL-TET	S	S	S	S	S	S	R	R
SUL-TET	S	S	S	S	S	S	R	R
SUL	S	S	S	S	S	S	R	S
SUL-TET	S	S	S	S	S	S	R	R
SUL	S	S	S	S	S	S	R	S
CLI-ERY-OXA-SUL-TET	I	S	R	R	S	R	R	R
CHL-CLI-ERY-OXA-SUL-TET	R	S	R	R	S	R	R	R
CLI-ERY-GEN-SUL-TET	I	S	R	R	R	S	R	R
SUL	S	S	S	S	S	S	R	S
CIP-CLI-OXA-SUL	S	R	R	S	I	R	R	I
CLI-ERY-OXA-SUL-TET	I	S	R	R	S	R	R	R
CLI-OXA-SUL	S	S	R	S	S	R	R	S

CHL-CLI-ERY-OXA-SUL-TET	R	S	R	R	I	R	R	R
CLI-ERY-OXA-SUL-TET	S	S	R	R	S	R	R	R
ERY-OXA-SUL	S	S	S	R	S	R	R	S
CHL-CLI-ERY-OXA-GEN-SUL-TET	R	S	R	R	R	R	R	R
CIP-ERY-OXA-SUL	S	R	S	R	S	R	R	S
ERY-OXA-SUL	S	S	S	R	S	R	R	S
ERY-OXA-SUL	S	S	S	R	S	R	R	S
ERY-OXA-SUL	S	S	S	R	S	R	R	S
ERY-OXA-SUL	S	S	S	R	S	R	R	S
ERY-OXA-SUL	S	S	S	R	S	R	R	S
ERY-OXA-SUL	S	S	S	R	S	R	R	S
ERY-OXA-SUL-TET	S	S	S	R	S	R	R	R
ERY-OXA-SUL	S	S	S	R	S	R	R	S
ERY-OXA-SUL	S	S	S	R	S	R	R	S
ERY-OXA-SUL	S	S	S	R	S	R	R	S
CLI-ERY-SUL	S	S	R	R	S	S	R	S
CHL-CIP-CLI-ERY-GEN-OXA-SUL-TET	R	R	R	R	R	R	R	R
CHL-CIP-CLI-ERY-GEN-OXA-SUL-TET	R	R	R	R	R	R	R	R
CHL-CIP-CLI-ERY-GEN-SUL-TET	R	R	R	R	R	S	R	R
CHL-CIP-CLI-ERY-GEN-SUL-TET	R	R	R	R	R	S	R	R
CHL-CIP-CLI-ERY-GEN-SUL-TET	R	R	R	R	R	S	R	R
CHL-CIP-CLI-ERY-GEN-SUL-TET	R	R	R	R	R	S	R	R
CHL-CIP-CLI-ERY-GEN-SUL-TET	R	R	R	R	R	S	R	R
CLI-ERY-OXA-SUL-TET	S	S	R	R	S	R	R	R
CHL-CIP-CLI-ERY-GEN-SUL-TET	R	R	R	R	R	S	R	R
CHL-CIP-CLI-ERY-GEN-SUL-TET	R	R	R	R	R	S	R	R
CHL-CIP-CLI-ERY-GEN-SUL-TET	R	R	R	R	R	S	R	R
CIP-CLI-ERY-GRN-SUL	S	R	R	R	R	S	R	S
CHL-CIP-CLI-ERY-GEN-OXA-SUL-TET	R	R	R	R	R	R	R	R
ERY-SUL-TET	S	S	S	R	S	S	R	R
CHL-CIP-CLI-ERY-GEN-OXA-SUL-TET	R	R	R	R	R	R	R	R
SUL	S	S	S	S	S	S	R	S
SUL	S	S	S	S	S	S	R	S
CHL-CIP-CLI-ERY-GEN-SUL-TET	R	R	R	R	R	S	R	R
CHL-CIP-CLI-ERY-GEN-SUL-TET	R	R	R	R	R	S	R	R
CHL-CIP-CLI-ERY-GEN-OXA-SUL-TET	R	R	R	R	R	R	R	R
CHL-CIP-CLI-ERY-GEN-SUL-TET	R	R	R	R	R	S	R	R
CHL-ERY-SUL-TET	R	S	S	R	S	S	R	R
CLI-ERY-SUL-TET	I	I	R	R	S	S	R	R
CHL-CIP-CLI-ERY-GEN-SUL-TET	R	R	R	R	R	S	R	R
CHL-CIP-CLI-ERY-GEN-OXA-SUL-TET	R	R	R	R	R	R	R	R
CHL-CIP-CLI-ERY-GEN-OXA-SUL-TET	R	8	R	R	R	R	R	R
CHL-CIP-CLI-ERY-OXA-SUL-TET	R	R	R	R	I	R	R	R
CHL-CIP-CLI-ERY-GEN-SUL-TET	R	8	R	R	R	S	R	R
SUL	S	I	S	S	S	S	R	S
SUL-TET	S	S	S	S	S	S	R	R
CLI-OXA-SUL-TET	S	S	R	S	I	R	R	R
SUL-TET	S	I	S	S	S	S	R	R

SUL-TET	S	S	S	S	S	S	R	R
ERY-SUL-TET	S	S	S	R	S	S	R	R
ERY-SUL-TET	S	S	S	R	S	S	R	R
ERY-SUL	S	S	S	R	S	S	R	R
CHL-CIP-CLI-ERY-SUL-TET	R	R	R	R	I	S	R	R
CHL-CIP-CLI-ERY-GEN-OXA-SUL-TET	R	R	R	R	R	R	R	R
CHL-CIP-CLI-ERY-GEN-OXA-SUL-TET	R	R	R	R	R	R	R	R
ERY-SUL-TET	S	S	S	R	S	S	R	R
CHL-CLI-ERY-GEN-SUL-TET	R	S	R	R	R	S	R	R
CHL-CIP-CLI-ERY-GEN-OXA-SUL-TET	R	R	R	R	R	R	R	R
ERY-SUL-TET	S	S	S	R	S	S	R	R
CLI-ERY-OXA-SUL	S	S	R	R	S	R	R	S
CHL-CIP-ERY-OXA-SUL-TET	R	R	S	R	S	R	R	R
ERY-SUL-TET	S	S	S	R	S	S	R	R
CHL-CIP-CLI-ERY-SUL-TET	R	R	R	R	S	S	R	R
CLI-ERY-SUL-TET	S	S	R	R	S	S	R	R
CLI-ERY-SUL-TET	S	S	R	R	S	S	R	R
CLI-ERY-SUL-TET	S	S	R	R	S	S	R	R
ERY-SUL-TET	S	S	S	R	S	S	R	R
SUL-TET	S	S	S	S	S	S	R	R
CHL-CIP-CLI-ERY-GEN-SUL-TET	R	R	R	R	R	S	R	R
CHL-CIP-CLI-ERY-GEN-OXA-SUL-TET	R	R	R	R	R	R	R	R
CHL-CLI-ERY-SUL-TET	R	S	R	R	I	S	R	R
CLI-ERY-GEN-SUL-TET	S	S	R	R	R	S	R	R
CLI-ERY-GEN-SUL-TET	I	I	R	R	R	S	R	R
ERY-SUL-TET	I	S	I	R	S	S	R	R
CHL-CIP-CLI-ERY-GEN-OXA-SUL-TET	R	R	R	R	R	R	R	R
CHL-CIP-CLI-ERY-GEN-SUL-TET	R	R	R	R	R	S	R	R
CHL-CLI-ERY-GEN-SUL-TET	R	S	R	R	R	S	R	R
CLI-ERY-OXA-SUL-TET	S	S	R	R	I	R	R	R
ERY-SUL-TET	S	S	S	R	S	S	R	R
SUL-TET	S	S	S	S	S	S	R	R

Figure S1

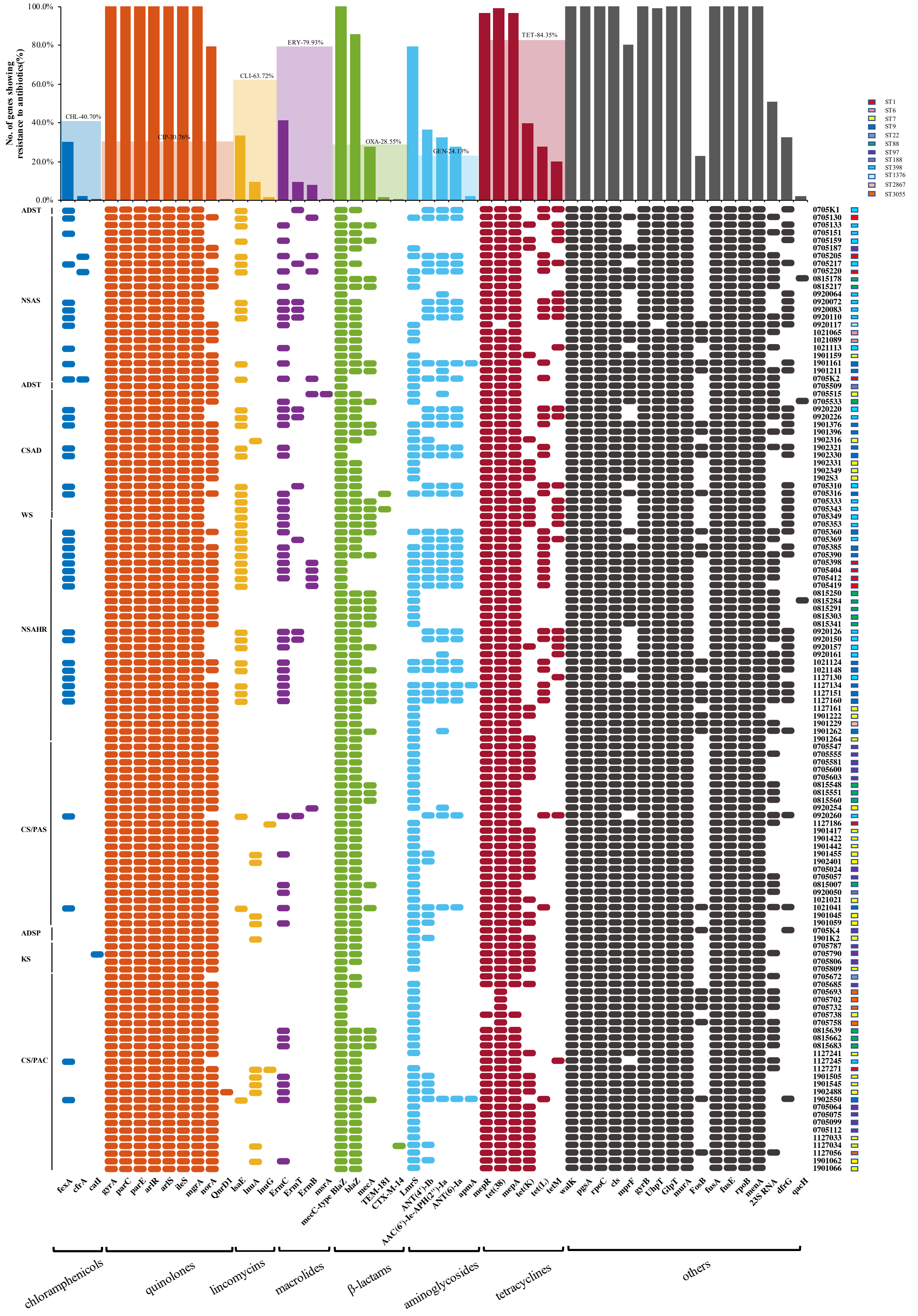

Figure S1. Heatmap showing the resistance genes in this study. Upper of the figure showing the number of genes (%) and the percentage of resistance to antibiotics (%). Different groups of resistance genes were color-coded.

Figure S2

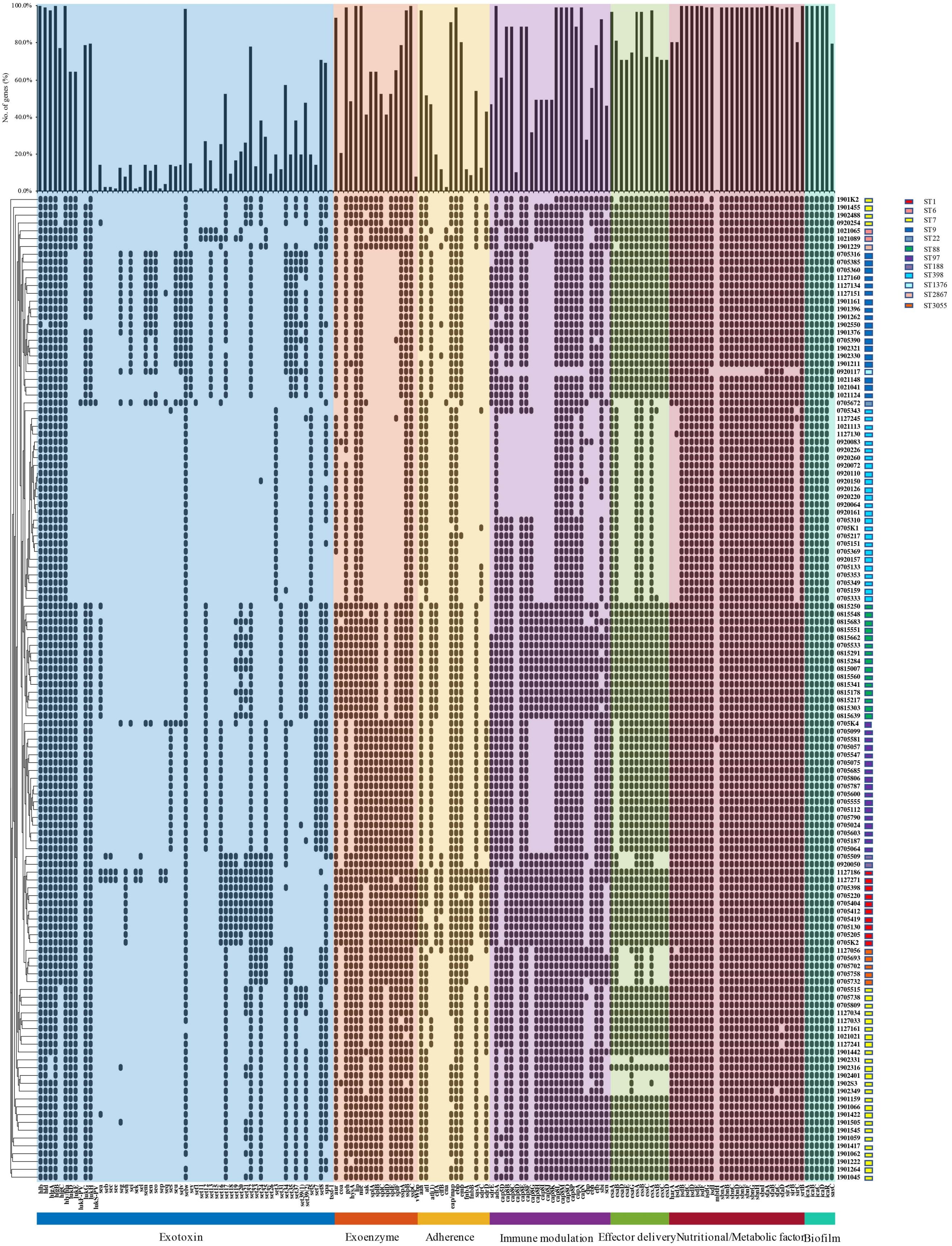

Figure S2. Heatmap showing the virulence genes in this study. Upper of the figure showing the number of genes (%), left side of the figure showing the ML tree of 126 *S.aureus* isolates with 1000 bootstraps, and the reference genome was *S. aureus* NCTC8325 (txid: CP000253.1). Different groups of virulence genes were color-coded.

Figure S3

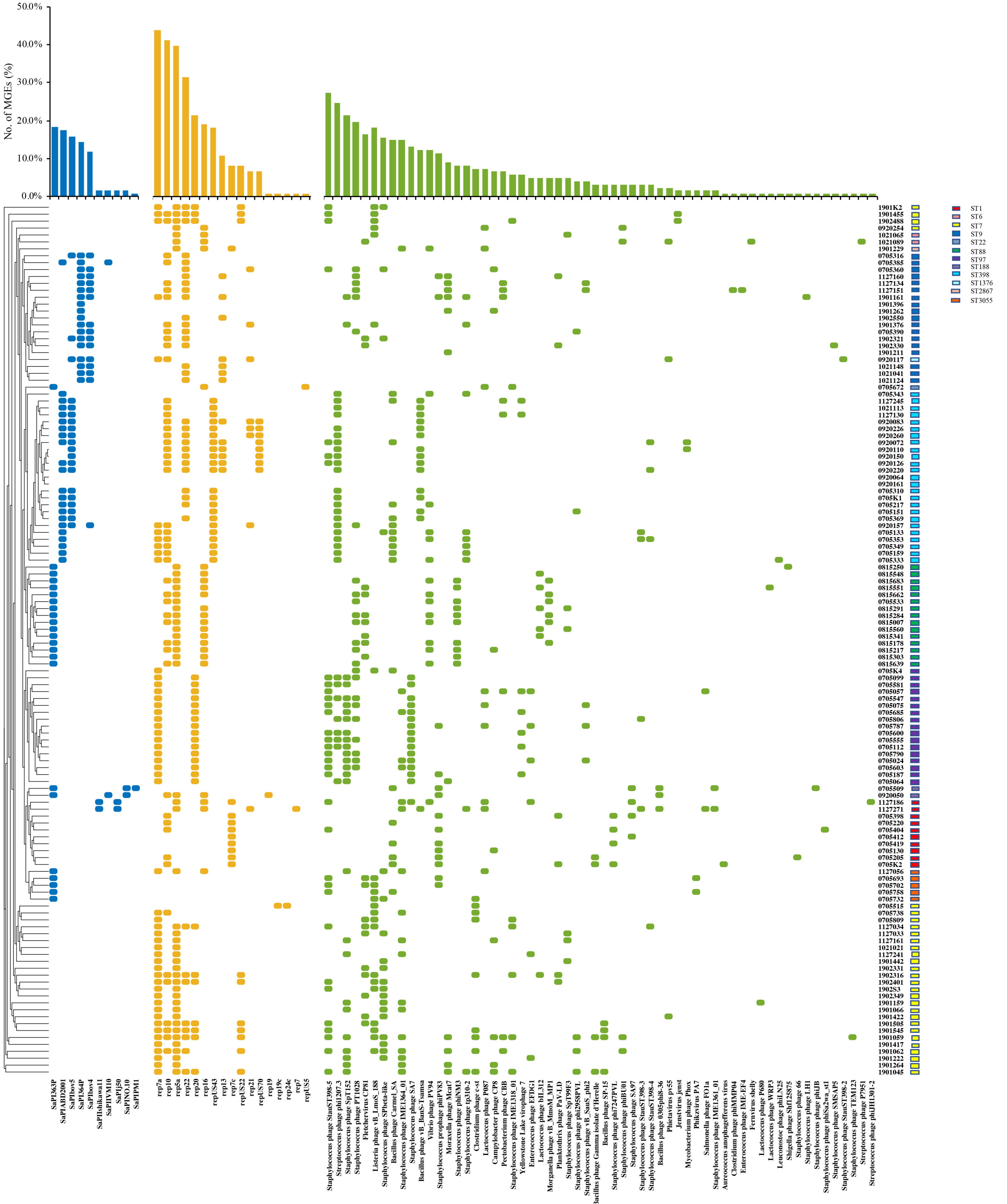

Figure S3. Mobile Genetic Elements profiles. Upper of the figure showing the number of MGEs (%), left side of the figure showing the ML tree of 126 *S. aureus* isolates with 1000 bootstraps, and the reference genome was *S. aureus* NCTC8325 (txid: CP000253.1). Different groups of MGEs were color-coded.

Figure S4

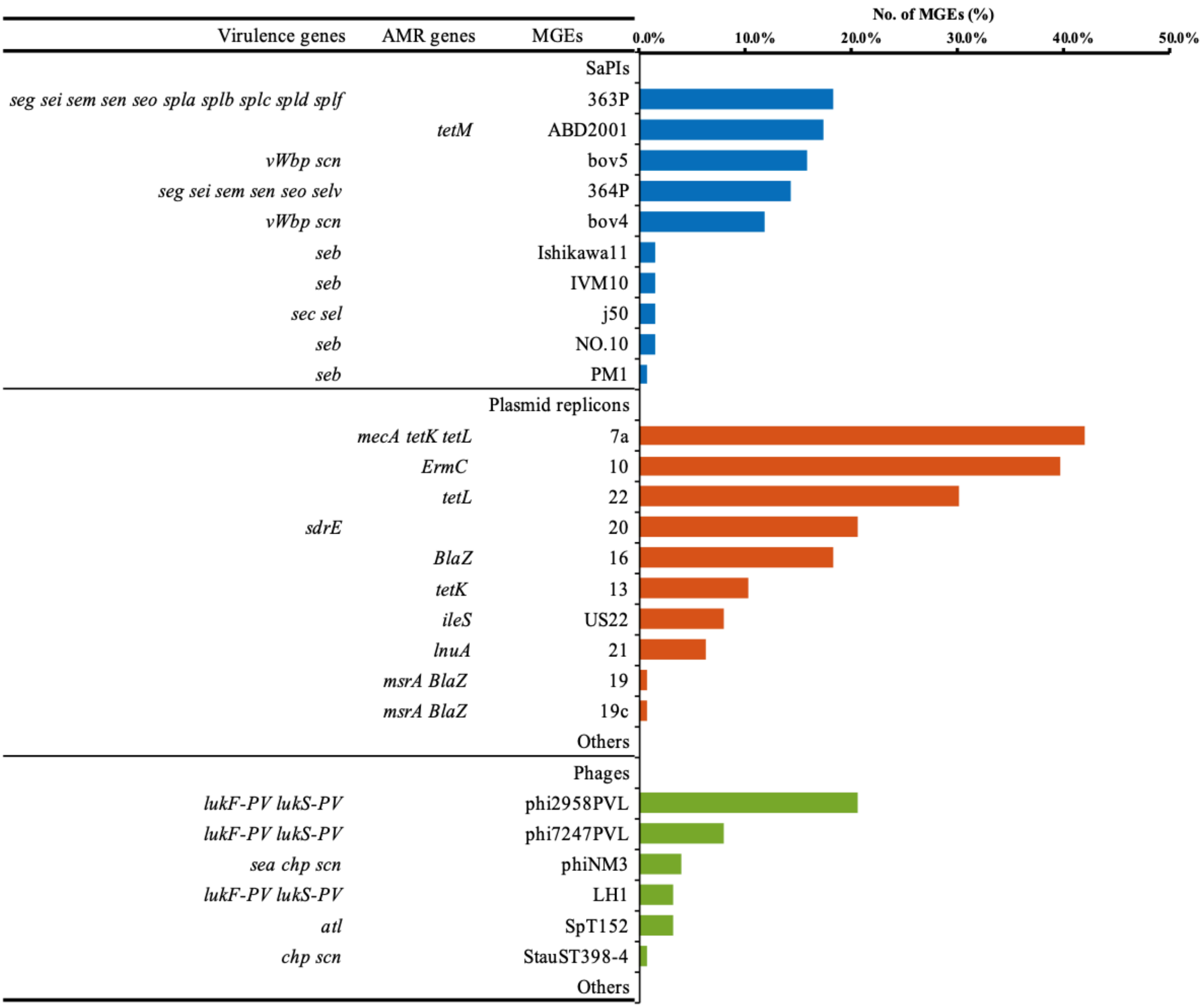

Figure S4. Distribution of MGEs and MGEs-encoded AMR genes and Virulence genes that identified in this study.
